# Proposing a multi-method phenomenological approach in exploring the perceived daily life experiences of people with dementia in their dementia care environments and immediate outdoor settings

**DOI:** 10.3389/frdem.2025.1502911

**Published:** 2025-03-31

**Authors:** Alexia Mercieca, Iain Scott, Catharine Ward Thompson, Heather Wilkinson

**Affiliations:** ^1^Faculty for the Built Environment, Department of Architecture and Urban Design, University of Malta, Msida, Malta; ^2^Edinburgh School of Architecture and Landscape Architecture, University of Edinburgh, Edinburgh, United Kingdom; ^3^School of Health in Social Science, ECRED Edinburgh Centre for Research on the Experience of Dementia, ACRC Academy, University of Edinburgh, Edinburgh, United Kingdom

**Keywords:** multi-methods, dementia design, architecture, dementia outdoors, enabling environments

## Abstract

The environment in this study is presented primarily drawing on the theoretical definition of home, and its experience and meaning to the individual with dementia, with an interest in access to outdoors. Notions of perception, cognitive image and affordance are central to the sense of home, and in turn the sense of self that this may inform and support. This theoretical framework informs the multi-method phenomenological approach proposed, through themes of spatial legibility, cultural appropriateness, fascination, user-centredness and personalisation. The novelty of the methodological toolkit lies in the incorporation of methods that have been traditionally used in research with people with dementia as the basis of the framework, but which are supplemented by additional layers developed from conventional architectural tools to create a more visual representation of the environmental experience. Despite its apparent complexity, the methodology yields a very clear and precise image of the person's presence in her surroundings, at once providing a location in space and time, her mood and engagement, as well as a layering of the affordances that may have informed her behavior. This method was developed as part of this research, and remains unique to it. Its innovation lies in the progression of the DCM tool, the integration of the notion of affordances and architectural mapping techniques to propose a holistic depiction of the care experience of people with dementia.

## Introduction

This article is based on an original piece of research undertaken as part of a PhD in Architecture at the University of Edinburgh, completed in 2023. The PhD research sought to explore the impact of dementia care environments through an exploration of the perceived daily life experiences of people with dementia in their respective dementia care environment. Dementia care environments in four different countries and three continents were included in this study.

The environment, and therefore the architecture and design of the space, holds valuable relevance to the care and treatment of people with dementia, particularly when operating as part of a broader cultural and social support system. A considerable body of evidence exists to support the potential impact of the physical environment on people with dementia, showing that appropriate design solutions may affect behavior, orientation, social function and overall wellbeing (Marquardt, et al. 2014, as cited in Easton and Ratcliffe, 2020). Zeisel (2006) argues that studying people with dementia in negotiating their immediate environment “offers a window into the critical role environments play in cognitive mapping, memory, and self-awareness because the brains of people with Alzheimer's have such an extreme need for support in these areas” (p. 370). Design is one of the more widely accepted non-pharmacological approaches toward improving the quality of life of persons experiencing cognitive decline residing within long-term care environments (Zeisel and Raia, 2000). People with dementia may indeed experience improved mood and wellbeing when treated in appropriately planned and designed environments; “They can develop both a sense of self and of belonging to a larger community of residents” (Zeisel and Raia, 2000, p. 5).

Probing a highly inclusive research strategy that challenges the inequalities experienced by people with dementia and seeks to obtain and document their own personal experiences (Wilkinson, 2002) is central to the study. This has led to the inherent need to develop a customized methodology to record the personal experience of each participant, thereby recognizing the uniqueness of each individual and validating their lived experience.

Informing the methodology is an exploration of the concept of home in its broader sense. It is the concept of home as a safe harbor that fosters relationships, nurtures a sense of self and supports personal development and wellbeing. It is the home as the host to our memories, both positive and sometimes negative. It is the notion of home that offers the opportunity to mold one's own habitat, to claim a place in the world (Cooper Marcus, 2006) and to assert oneself within a community. As attested through the phrase “*I want to go home*,” undoubtedly the most common phrase recorded during the duration of the research study, the sense of home is rife even in the face of cognitive decline, and the warm feeling of “being at home” is still highly sought after through the several phases of the dementia journey (Cerejeira et al., 2012).

A quality care environment is believed to be one where the physical context can foster a sense of home for the person residing within it. The study recognizes the vast body of literature available on the topic of appropriate care environments for people with dementia, including accepted design guidelines and assessment tools for dementia care environments (Bowes and Dawson, 2019). In acknowledging the value and need for such standards, it proposes a reading of the environment through the experience of the person with dementia. This phenomenological approach is developed around notions of personhood and identity, as well as inclusion within the residential community, as potentially being supported by the physical setting.

### Research rationale

This research aims to explore the physical extents of the care environment as it may be experienced by the residents, thereby placing people with dementia at the center of the research. People with dementia are fundamental to the study due to their sensitivity to their immediate environment, and the uninhibited way in which they respond to such surroundings, while also being highly vulnerable to them. In their subjective individuality, people with dementia represent every person's yearning to live in an environment that is supportive, empathetic and responsive to their needs.

Motivation: the research is motivated by a need for more responsive care environments for people with dementia, who are less likely to be able to express their needs or modify their surroundings in a way that suits them, and safe in the knowledge that an environment that is suitable for people with dementia, is beneficial to all users. The research therefore seeks a methodology that may assess the diverse elements in the care environments and their outdoor spaces in terms of the behavior they afford residents with dementia, to ascertain which may be more conducive to positive wellbeing.

Audience: the primary audience is interdisciplinary researchers with backgrounds in architectural design and theory, health sciences and environment-behavior studies, dementia and aging, as well academics across the disciplines of architecture and healthcare. The secondary audience is architects, designers and care-practitioners who are involved in the design and management of new care environments for people with dementia and who may have an interest in more person-centered residences. The broader outreach would be toward policy-makers, in the hope that design that is more conscious of the realities of people with dementia becomes more available to the people who need it.

Impact: for people with dementia, the research seeks to emphasize the design of long-term care environments that are familiar and culturally responsive while supporting the individuals residing within, safeguarding their autonomy and their freedom. In the field of architecture and design, the research offers an opportunity to reconsider care environments that are more domestic and challenge the traditional clinical care setting. By providing the opportunity for legibility of real-life dementia care scenarios in a highly graphical manner, the research attempts to make the care experience accessible to a broader audience, thereby raising much needed awareness.

The following is the objective that the study seeks to achieve:

To conceive a methodological structure that evaluates mood, engagement and comprehensive wellbeing of persons with dementia, in terms of interactions with the intrinsic qualities of their immediate environment, both physical and non-physical, indoor and outdoor.

The following is the research question that the study seeks to address:

How may available research methods be adapted and developed to enhance knowledge of the care environment experience as perceived by residents with dementia?

### Introducing the method

The multi-method phenomenological approach proposed here was designed in an iterative manner as the research study developed and sought to be the first method of its type that documents the behavior and experience of people with dementia through the use of architectural research tools. The methodology is novel in that it incorporates a unique set of elements that provide the reader with a window onto the reality of the dementia care environment as experienced by the person with dementia. It seeks to be accessible to a broader audience, beyond the healthcare realm, including the architecture community through its design language, and the public through its clear, straightforward graphics, thereby propagating the experience of the dementia care environment further.

## The theoretical framework informing the methodology

The research methodology must essentially be grounded in a theoretical framework that is understandable (Wilkinson, 2004) and responds to the research questions set out. The following theoretical framework sets the basis for the research methodology developed, where the iterative process followed during the development and refinement of the method is integral to the final methodology adopted.

The nature of this approach is deeply rooted in qualitative research, drawing its typical focus on contemporary circumstances that are both social and cultural (Groat and Wang, 2013), and its ability to access areas of research that are inaccessible by other methods of inquiry (Sumathipala et al., 2004). Denzin and Lincoln (1998, as cited in Groat and Wang, 2013) give the following definition of qualitative research:

“Qualitative research is multi-method in focus, involving an interpretive, naturalistic approach to its subject matter. This means that qualitative researchers study things in their natural settings, attempting to make sense of, or interpret phenomena in terms of the meanings people bring to them. Qualitative research involves the studied use and collection of a variety of empirical materials.” (p. 218)

Qualitative research is also highly inductive, following a process of open-ended questions, exploration, re-thinking the questions and proposing a final set of questions in an iterative, non-linear process that thereby reflects an increased understanding of the problem (Creswell, 2007).

Groat and Wang (2013) proposed five key components of qualitative research, which have also guided the development of the research methodology for this study:

1. **An emphasis on natural settings**. A natural setting is one wherein the objects under investigation exist as they typically would in everyday life (Groat and Wang, 2013). In the case of this study, the in-depth observations carried out over several time periods, within different care settings, were essential tactics in studying people within their domestic care context, while studying the context itself in its natural state. Sound knowledge of the context, as expressed through its rituals and culture is also vital in deciphering the person's role within the group context.
2. **A focus on interpretation and meaning**. Qualitative research work is grounded in the empirical realities of observations and interviews with participants but is also very much dependent on the role of the researcher in interpreting and relating the data. This is defined precisely by Cuff (1991, as cited in Groat and Wang, 2013): “Philosophically, what I value... is [a] rejection of positivist notions of the social world, embracing interpretation, meaning in context, interaction, and the quality of commonplace” (p. 215). The value of interpretation and meaning is even more valuable in this research project due to the limited interaction allowed under the ethics guidelines governing the research to safeguard the vulnerability of the residents. Creativity in accessing and interpreting the participants' behavior is essential when working with this user group. The subjectivity of my personal interpretation and meaning, as the main and only researcher on this project, is also extended to the detailed coding and presentation process followed, which will be discussed in further detail later in this paper.
3. **A focus on how the respondents make sense of their own circumstances**. Groat and Wang (2013) suggest that this would entail different methods to present a holistic image of the phenomenon or setting under investigation, from the point of view of the participants themselves. In the context of people with dementia in a formal care setting, this signifies added complexity due to the restrictions on verbal engagement. Moreover, a number of residents had reached a stage in their dementia journey where they exhibited difficulty in expressing themselves verbally, or were altogether non-verbal, however they could still communicate through their behavior. Knowledge and insight into the behavior of people with dementia was therefore crucial in interpreting and giving meaning to the behavior, or absence of certain behaviors, observed.
4. **The use of multiple tactics**. Groat and Wang (2013) describe qualitative research as being characteristic of several practices that are brought together to address a specific problem or situation. The tactics employed should be particular to the context being studied and pertinent to the research questions being asked. The multiple tactics brought together into the research strategy for this study are discussed further on in this paper.
5. **Significance of inductive logic**. An iterative process is generally common to a qualitative study (Creswell, 2007; Groat and Wang, 2013). It is common for the research question(s) to be scrutinized, tweaked and revisited throughout the development of the research, engagement with participants and gathering of data, giving the researcher the opportunity to test and embrace emerging insights (Groat and Wang, 2013). This organic evolution was also very significant for the research project as it developed in this case, wherein the struggle with the extent to which I could plan and control the process was very real. The realization that a more open attitude toward new or unexpected events was more conducive toward fresh ways of collecting and interpreting the data in meaningful ways, provided a major shift in the quality of the methodology developed. Such iterative processes are generally more labor and time intensive due to the sheer volume of data that is analyzed throughout the progress.

### Situating the study in the contemporary research continuum

**Figure 1:**
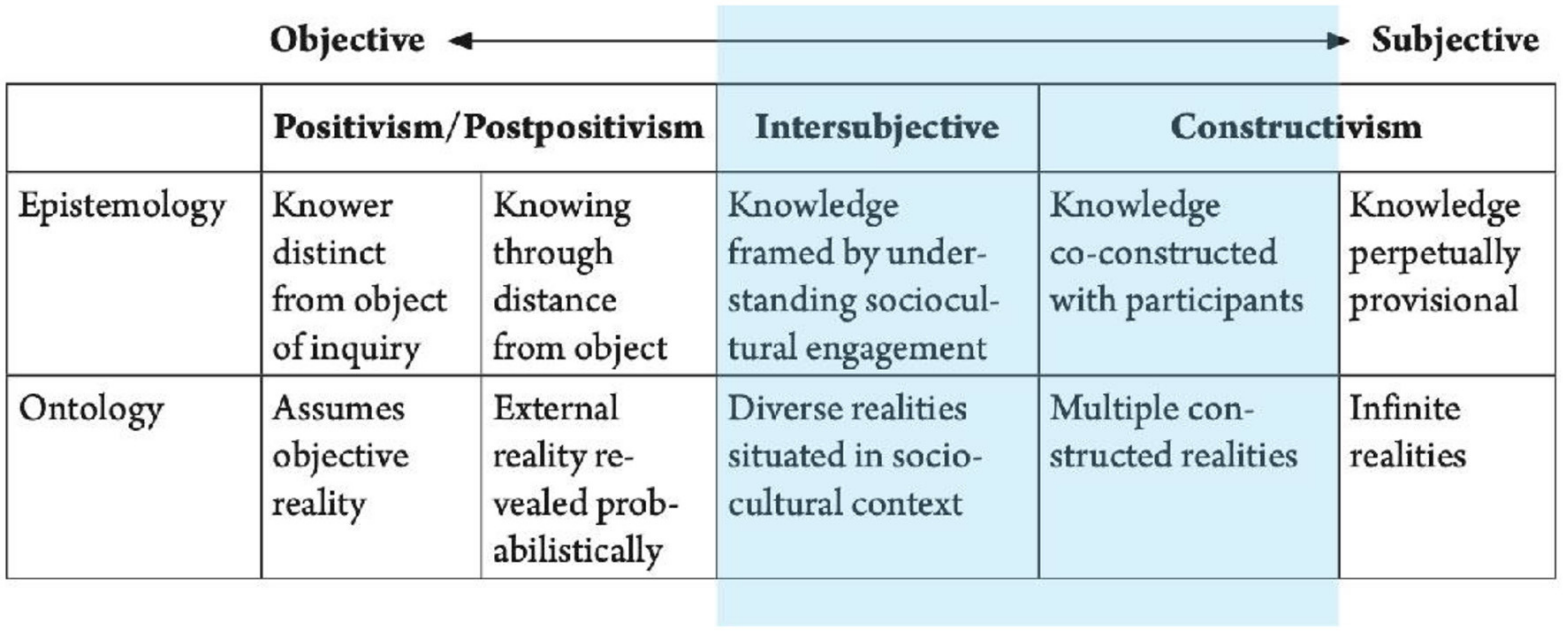
Continuum of research paradigms. Source: Groat and Wang (2013, p. 67) (Adapted from Mugerauer, 1995; Guba and Lincoln, 1998; Teddlie and Tashakorri, 2009; Mertens, 2010).

Groat and Wang (2013) have responded to the traditional dichotomous epistemological models of research analysis (qualitative and quantitative) with an alternative continuum of research paradigms, based on their review of several paradigms developed by a broad range of authors. Their model proposes three primary epistemological positions, across which they acknowledge the possibility of multiple epistemological and ontological positions (Groat and Wang, 2013). Figure 1 shows this continuum, which is bounded by constructivism at one end and the positivist/postpositivist tradition at the other end. Due the breadth of the multiple schools of thought represented in the central section, and the lack of a widely accepted label, Groat and Wang (2013) have suggested the use of the term intersubjective, “to reflect its interstitial position between the positivist emphasis on objectivity and the constructivist emphasis on subjectivity” (p. 76). This continuum is reflective of the contemporary fluidity that exists in the characterization of different epistemological traditions and the differences between different academic disciplines (Groat and Wang, 2013). Moreover, it is significant of the intrinsic multidisciplinary nature of architecture, as a profession and as a discipline, that ranges from highly technical scenarios to cultural contexts and historical enquiries (Groat and Wang, 2013).

This study was undertaken across the threshold between intersubjectivity and constructivism, as highlighted in blue on the same Figure 1, and described in further detail here to contextualize the proposed methodology in the research milieu, particularly as defined in the continuum proposed by Groat and Wang (2013).

The essence of the **intersubjective** paradigm states that the world is understood through sociocultural engagement, and, “[o]ntologically, it assumes, that although there are multiple diverse viewpoints regarding sociocultural realities, it is nevertheless possible to achieve shared understandings of those realities” (Groat and Wang, 2013, p. 78). Therefore, rather than striving to achieve objective accord, values and meaning are established in the framing of the research goals and in the contextual interpretation of the results (Teddlie and Tashakorri, 2009, as cited in Groat and Wang, 2013).

**Constructivism** exists at the subjective end of the continuum, however for the sake of this research, the focus is placed on the section closer to the intersubjective reality, which we are hereby defining as the intersubjective-constructivist threshold. Denzin and Lincoln (2008, as cited in Groat and Wang, 2013) define this constructivism paradigm as, “entailing a ‘relativist' ontology, whereby multiple realities are understood as being socially constructed” (p.78). A constructivist approach would be one that elicits deep insights and interpretations of an environment or settings from the point of view of the individual experiencing that environment (Groat and Wang, 2013). In an ideal scenario, knowledge emerges from the co-creation of understandings of the situation or context under investigation, between the researcher and participant(s) (Groat and Wang, 2013), however this was not always an option in the case of this research.

The main part of the research methodology, carried out within the dementia care environments, is constructivist in the way it was open to the reception of broad data from inception. The pilot study was specifically devised for this reason, and was carried out following a more constructivist approach, to ensure most scenarios possible can indeed be captured and interpreted through the analysis. The study also adopts the interstitial stage in the way it managed the data received, to make the results more communicable and accessible to a broader audience, while maintaining that each experience is unique, not only to the individual, but specifically to that individual in that environment, at that point in time during her dementia journey. It is also for this reason that each site, and therefore each care environment, should be considered uniquely, and not in comparison to the other sites under investigation. This ensures that the subjectivity of each experience is interpreted in the socio-cultural context in which it was created, whilst also acknowledging the multiple constructed realities that co-exist within the seemingly identical context. The person's engagement within the social context and with the surroundings therefore becomes the only tool toward the generation of knowledge, where depth is achieved by exploring multiple interpretations of the data collected and interpreting it against a strong theoretical context.

## Reviewing previous studies, methodologies and frameworks

Several studies were reviewed as part of the general research phase, and have been included here following an iterative process, which reflected the general process followed also through the research methodology strategy as discussed further on. The process started with the review of the main categories identified: care practice studies, physical environment studies and person-centered environment-based studies. These served to provide the broader knowledge required to pursue the fieldwork. Following the pilot study and the analysis of the first set of results obtained, there was a felt need to review further studies to enhance the methodological review, in response to the themes emergent from these pilot results. These were specifically around the themes of the theory of affordances, in terms of how a person's behavior is a function of that which is offered by her immediate environment, and phenomenological approaches specific to the experience of people with dementia, relating to a better understanding of the experiential value of different scenarios within the care environment. The methodological contextualization of these two categories is discussed in the next section.

The primary, and more general categories identified as relevant to this study are discussed further below.

**Figure 2:**
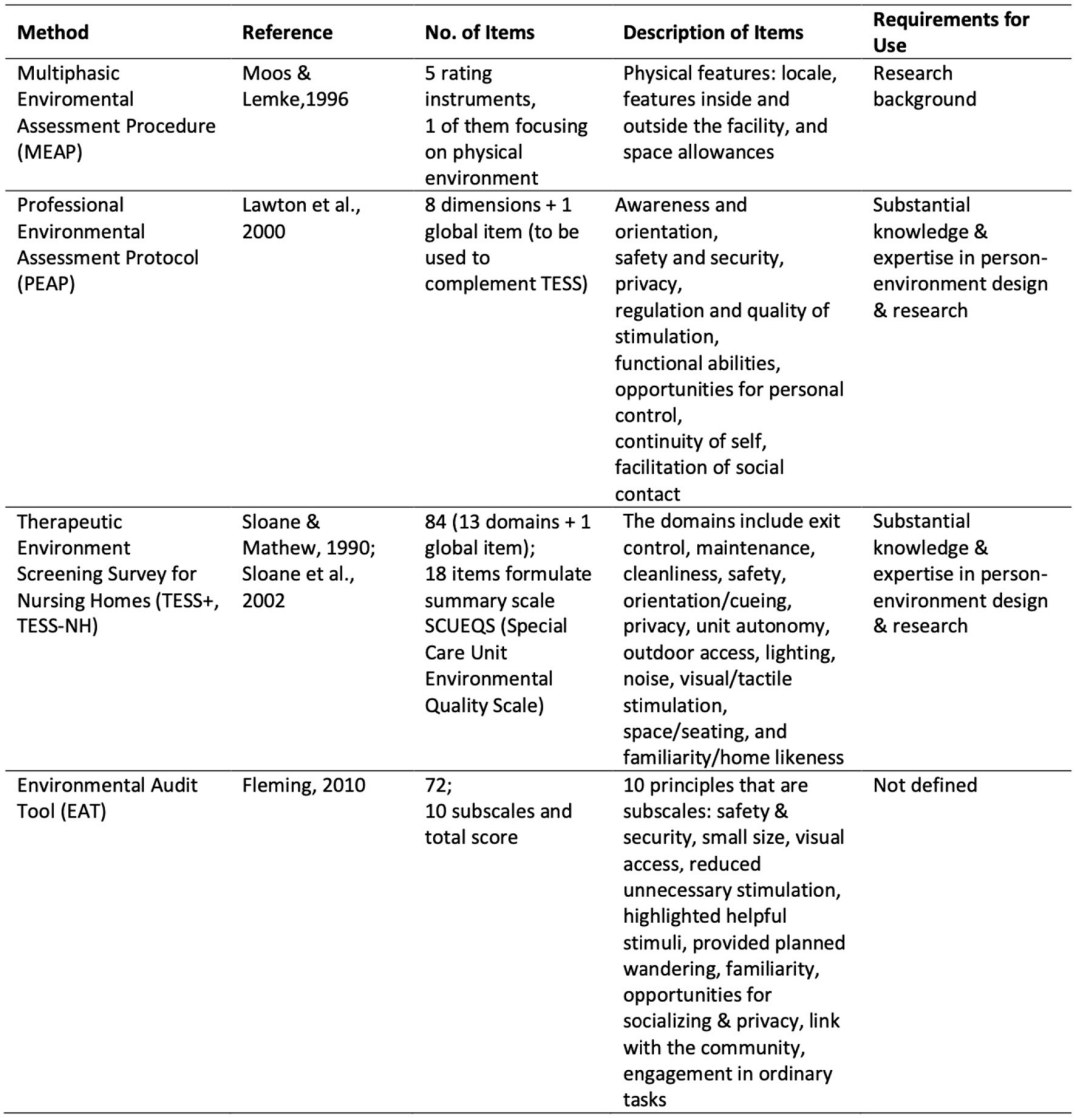
Four internationally recognized assessment methods for residential care environments. Source: Topo et al. (2012, p. 122).

1. **Care practice studies** are those that focus specifically on the wellbeing of the person with dementia from the point of view of the quality of the care provided, with little to no regard for the built environment and its potential effect on the person with dementia or her carer(s) (Kitwood, 2019).
2. **Physical environment studies** relevant to this research study are those that relate to the environments of residential care settings. This research focused on the physical characteristics of the care environment and how these affect the wellbeing of the person with dementia residing within (Zeisel et al., 2003; Chalfont, 2008; Dementia Services Development Centre, 2008; Bowes and Dawson, 2019). Much of this literature draws from assessment tools which are based on checklists of quantitative measurements (Topo et al., 2012), the most common of which (MEAP, PEAP, TESS+, TESS-NH, and EAT) have been summarized in Figure 2. Such tools are often preventive in character, proposing actions to reduce negative factors (Kuliga et al., 2021) rather than focusing on the potential for supporting positive factors of the person's remaining abilities.

A review of a number of recent studies on the physical characteristics of care homes and the ways in which these were deemed to respond to the needs of the people with dementia resident within revealed that smaller scale care environments that provide a homelike setting are generally preferred to large-scale institutional settings as they are generally believed to provide more positive stimulation, resulting in less withdrawn behavior and improved quality of life (Lee et al., 2021). The most common themes to emerge as conducive to an improved experience of the care environment are appropriately individualized spaces and familiar physical environments (Bowes and Dawson, 2019). The kitchen is broadly regarded as the heart of the house, providing a sense of familiarity, as well as promoting engagement through simple rituals and daily routines which the residents would have been well accustomed to prior to the onset of their dementia. Marsden et al. (2001) argued that fully functioning kitchens that are part of the main food service provision of the care environment are more valuable to the residents than kitchens that are reserved exclusively to catering activities. They provide a homelike environment and offer cues for residents' known behaviors in preparing a cup of tea or rinsing dishes independently. Moreover, Chalfont (2008) argues that people's food habits and choices are a means of maintaining their own cultural identity and sense of self, therefore further emphasizing the highly positive behavior that may emerge from the inclusion of an appropriately designed kitchen. A number of studies that look at the use of the kitchen in the care context go to great lengths to describe the setup observed, how people interact across it, with their surroundings, with one another, and with the staff (Hyden, 2014). This study, while recognizing the fundamental relevance of the kitchen, proposes architectural tools to aid with the depiction of the given scenario, as well as the intricate exchanges afforded within.

A household-style care layout, designed around a communal kitchen and living space, is considered to contribute to communication and interaction between the residents, as well as with the staff and family members (Morgan-Brown and Chard, 2014). Also, a familiar, home-like environment was shown to be more conducive toward self-initiated interaction and communication, with less input and prompting from staff members required (Morgan-Brown and Chard, 2014). This also links to the level of environmental press present in the environment (Nahemow and Powell Lawton, 1973), and the likelihood that a more homelike environment includes more cues and recognizable elements that invite the person to interact and participate, in group or alone. This stimulation in turn optimizes the observed quality of the behavior and the perceived sense of competence (Nahemow and Powell Lawton, 1973). Moreover, Morgan-Brown and Chard (2014) argued that the more homelike environments also provided for more privacy, and meaningful ways of being alone. This is an important viewpoint, in that many studies tend to place more value on conviviality and communal outputs than on privacy and seeking wellbeing alone or independently. Being alone in a positive way is also part of the reality of the domestic experience, and as such ought to be afforded also through the design of the care environment. This study places particular importance on this, both indoors and outdoors, depicting spaces for privacy more explicitly as spaces for potential positive levels of wellbeing. It responds to the felt need for research that critically reflects on environmental design principles, intervention tools and strategies (Kuliga et al., 2021) toward the amelioration of the environmental experience.

**Person-centered environment-based studies** are those that attempt to draw the person with dementia into the discourse on the physical environment. Such studies are more recent, driven by a shift that places more value on the person with dementia and her experience of the dementia care environment (Chalfont, 2008; Bowes and Dawson, 2019), as opposed to relying on accounts by care staff and family members (Ory Hernandez, 2012; Olsson et al., 2013). Such literature proposes the design of care environments that are more sensitized to the physical, psychological and cognitive changes experienced by the person with dementia (Judd et al., 1998). Research strategies that are creative in their methods and person-centered in their goals have the potential to really change the social practice of research (Webb et al., 2020).

Literature relating to the use of outdoor spaces and respective guidelines informing a more positive experience of the outdoor space (Tyson, 2002; Zeisel, 2006; Marshall and Pollock, 2012; Rodiek and Schwarz, 2012) is also very relevant here. Chalfont (2008) argues that there is still a felt need for “further evidence-based research showing which design elements particularly facilitate use of outdoor areas and the benefits people with dementia can receive” (p. 87). This is also essentially related to the ongoing recognition of the more extensive effects of walking on our lives and holistic wellbeing (Chalfont, 2008). This has in turn inspired further research and creativity in exploring how further movement and walking can be afforded within the context of the dementia care environment (Bennett, 2006). This broader understanding of walking has the potential of affecting “how both the physical and social aspects of care environments are conceived and designed” (p. 97).

Walking, together with issues pertaining to accessibility, freedom, privacy, and the health and wellbeing of the person with dementia who walks (Lai and Arthur, 2003; Marshall, 2006; Marshall and Pollock, 2012), are central to the development of this research strategy.

### Situating the theory of affordances in the context of the methodology

Gibson (1979/2015), in his definition of affordances, suggests that “what we perceive when we look at objects are their affordances, not their qualities” (p. 3). Therefore, as humans we are attracted to what an object affords us (Gibson, 1979/2015), and this is in turn exhibited through our behavioral response. He further argues that as an invariant combination of variables, “[t]hose features of a thing are noticed which distinguish it from other things that it is not—but not *all* the features that distinguish it from *everything* that it is not” (Gibson, 1966b, as cited in Gibson, 1979/2015, p. 125). Therefore, an affordance is not all-encompassing and universal, and it is not quantifiable objectively. Kytta (2004) offers a beautiful definition for this rapport: “The environment has to provide something that the individual can perceive as offering the potential for activity, but the perception emerges only when the different characteristics of the individual, such as his or her physical dimensions and abilities, social needs and personal intentions, are matched with the environmental features” (p. 181). Given that the affordance exists at the interface between the person and the environment, it has the potential to extend beyond the world of movable objects to include perceivable cultural, emotional and social opportunities available.

An affordance generates different responses in different persons, notably, it engenders different responses from the same person when set in different contexts. This is essential to the choice of affordance perception as a key tool of analysis for this research, as opposed to the analysis of the objective qualities of an object or element within the environment, as would be the case of a traditional checklist for dementia care environments.

**Figure 3:**
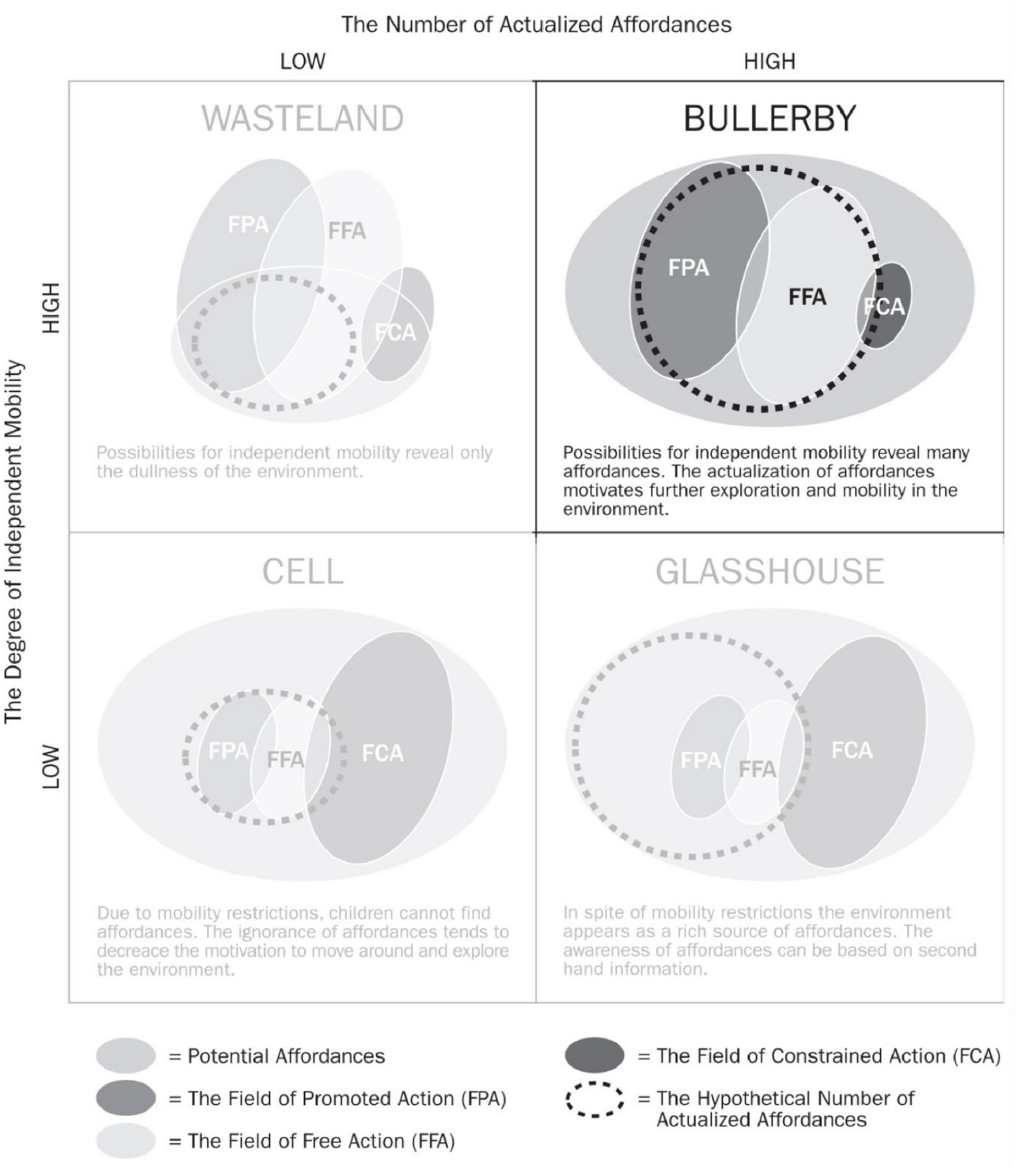
A model for describing four hypothetical types of environments that emerge from the co-variation of children's independent mobility and the number of actualised affordances. Source: Kontos (2004, p. 183), adjusted by author to focus on Bullerby example.

Kytta's (2004) study on the correlation between the number of actualised affordances and the degree of children's independent mobility across different environments suggests that affordances are not only perceived but can also be shaped. In her Bullerby example, reproduced in Figure 3, she concluded that independent mobility in children reveals many affordances which in turn motivate further exploration and movement through the environment (Kytta, 2004), in a sort of virtuous cycle.

Albeit deriving from research with children, the perceptive value of the study is deemed applicable to all age groups. Kytta's (2004) findings inform this research strategy in two ways:

1. The comparison between scenarios affording high and low degrees of independent mobility is paralleled with care environments possibly affording more or less independent mobility. This leads to the proposition that independent mobility is not a function dependent exclusively on the person's physical strength, but also one that is afforded in a relationship with the physical context.
2. Environments offering limited affordances lead to lack of interest and motivation, regardless of the degree of independent mobility. This advances the premise that the seeming detachment and lack of interest or participation of a person with dementia, may not be solely derived from the effects of the dementia, but is also afforded in relation to the environment.

It is worth noting that in the reality of a person with dementia, the notion of agency is central in the way affordances are perceived and actualised, particularly as the person becomes more dependent and may necessitate the intervention of care staff to mediate affordances for her (Topo et al., 2012). In the same way, the typology of the affordances may require adjustment to suit the changes experienced by the person due to the effects of the dementia. People with dementia would, at times, also exhibit difficulty in perceiving common objects and distances, therefore the possibility of false affordances may also arise (Topo et al., 2012).

### Situating phenomenological approaches in the context of the methodology

The phenomenological approach toward the understanding of human experience followed here is derived from the notion of acquired worlds as described by Merleau-Ponty (1945/2012) and the extent to which a person's sense of experience is generally governed by acquired concepts and judgements in a seemingly automatic and subconscious process (Merleau-Ponty, 1945/2012; Lynch, 1960; Ballantyne, 2007).

For a person with dementia, it was traditionally believed that access to such acquired worlds became more challenging with progression of the disease and loss of cognition (Kontos, 2004). However, affordance perception and selfhood have been shown to transcend cognition and memory loss, thereby demonstrating that selfhood “persists even with severe dementia, because it is an embodied dimension of human existence” (Kontos, 2004, p. 829). Selfhood is inextricably linked to the agency and status of the person, defining her individuality (Kontos, 2004; Kontos et al., 2017), which is in turn fundamental to the person's roles and freedoms, and the enjoyment of such rights.

In the context of the care environment, Kontos (2004) defines the agential powers of a person with dementia as apparent in the awareness of a person's surroundings, her engagement with the world, coherent interaction with emphasis on purpose and meaning. This extends to the person's intentionality in her movement and the way she navigates through a given environment (Kontos, 2004) which follows on from the definition of movement as a function of perception and is central to orientation and orienting oneself in a given context. Engagement with the world is in turn informed by that which is afforded, in a relationship between the reality of the individual and that which the surroundings may offer, and central to the methodology of this study.

The application of a phenomenological approach is therefore essential in situating the concept of home from the point of view of the person with dementia, with a focus on their competencies and individuality, an approach which is considered rare in studies concerning people with dementia (McColgan, 2005; Orulv, 2010). In a setting wherein the person has experienced several losses, including losses of home and physical objects, as well as losses of role and privacy (Orulv, 2010), the sense of familiarity and belonging engendered by the feeling of being at home is highly valuable.

In a study by Zingmark et al. (as cited in Orulv, 2010), the experience of being present, of being related to, or part of a larger whole, was significant in feeling at home for people with dementia, who often expressed the need to leave and get away when they felt lonely or abandoned. The possibility of participating and experiencing a sense of belonging with the staff and residents in the household has also been shown to foster a sense of involvement and collaboration (Hyden, 2014), which is also conducive toward a positive experience of the care environment. Participation may be simply in the form of sitting, being empowered with the choice of where to sit (McColgan, 2005) and following the general happenings in the care environment or just watching the world go by.

In supporting such constructive experiences, the design of the care environment and the model of care must follow the same narrative (Orulv, 2010), to avoid contradictory instances that may lead to confusion, frustration and disorientation. “Homeliness [is] fragile” (Orulv, 2010, p. 25), particularly in a care environment that may be very far from the person's perceived image of home.

In the context of the symptoms of dementia, the phenomenological approach proposed seeks to place the person at the center and design a legibility of the care environment from the point of view of the person with dementia and her experience of her immediate surroundings.

## Research strategy

“*We cannot separate what people do from where they do it.”*

Chalfont, 2008, p. 151

The theoretical context for the research strategy is pinned around the idea that, as humans are indeed a product of their environment, they become even more dependent on their surroundings and sensitized to the elements within it with the onset of dementia and progressive cognitive decline. Such person-environment interactions hold more significance as individual competence decreases with age, physical or psychological impairment. The research strategy sought to propose a novel way of recognizing these individual responses by analyzing the behavior of the person with dementia as a function of what her immediate environment affords her, toward an improved sense of wellbeing.

The first and main part of the research strategy was the development of a toolkit for assessing the role of dementia care environments in supporting residents with dementia through their daily activities within the care setting. The novelty of the toolkit lies in the inclusion of methods that have been traditionally used in research with people with dementia as the basis of the framework, that are supplemented by additional layers developed from architectural research tools to create a more visual representation of the environmental experience. Despite its apparent complexity, the methodology yields a very clear and concise image of the person's presence in her surroundings, at once providing a location in space and time, her mood and engagement, as well as a layering of the affordances that may have informed her behavior. This method was developed as part of this research and remains unique to it. Its innovation lies in the progression of the DCM (Dementia Care Mapping) tool (University of Bradford, 2016) and the integration of the notion of affordances (Topo et al., 2012) and architectural mapping techniques (Zeisel et al., 2003) to propose a holistic depiction of the care experience with particular interest in gardens and outdoor areas (Rappe et al., 2006).

The toolkit is the result of a highly iterative process that was developed following several visits to dementia care environments in Scotland and Malta, informal observations of residents with dementia in these environments and conversations with care staff in the respective care settings. This led to the development of the draft strategy, which was first applied during a pilot project held in a private care home in Malta. Following the collection of data and the first attempt at analyzing it, it was immediately obvious that further layers were required to really express the richness of the data and multiple facets of the person's care experience that this exposed. The users' need for connection with the outdoors was immediately noted and was assigned added importance in the next iteration of the model. This in turn informed the selection of fieldwork sites, across which the model was developed and tested, based on their connection with their immediate outdoor settings. The criteria for selection was for a residential care environment that hosts people who have received a dementia diagnosis in a care unit that affords connection to the immediate outdoor environment. The sites were selected based on the frequency of their appearance in literature, online presence and promotion as best practice care environments for people with dementia in their respective countries. The care environments wherein the research was carried out were based in Manchester (UK), Sydney (Australia), Fujisawa (Japan) and Luqa (Malta).

Further tools were considered, including the notion of affordances, particularly in the analysis and coding of behavior, and implemented in the updated version of the strategy that was applied to the first fieldwork site. Following this fieldwork experience and the larger volume of data that emerged, further iterations to the strategy were made in order to manage, analyse and present the data in a way that really expresses the essence of the experience of the person with dementia in her care environment. Further unique events that happened in one or more of the fieldwork sites led to the reconsideration of the method followed, informing further tweaks to the strategy and re-assessment of the data as a whole to ensure the best version of the strategy was put forward and applied. The research strategy for this part of the fieldwork grew considerably in complexity as further data was collected, prompting re-evaluation of the research objectives and questions, and accounting for a major part of the research thesis itself. In all, apart from minor tweaks, five full iterations of the model were created through its application in different care environments.

The toolkit being presented here is the result of this process and the final version that was applied across the fieldwork sites in the larger PhD study.

The toolkit is composed of four distinct tools as follows:

Tool 1—Behavior category codes charts.
Tool 2—Response charts.
Tool 3—Travel route maps.
Tool 4—Cultural setting diagrams.

The tools can exist separately but are ideally applied consecutively toward a holistic depiction of the person-environment dynamics under analysis in each care setting. One environment can host several such dynamics, as emerging from the interaction between the individuals and their environment. The environment encompasses the breadth of perceivable elements, both indoors and outdoors.

The behavior emerging from the person-environment interface is analyzed in the context of the “experiential paradigm” as discussed by Southwell (2004) and developed by pioneering researchers Cullen (1961), Lynch (1960) and Alexander (1977) whose approaches in landscape assessment and perception research had practical relevance in the approach to design, besides their propagation of knowledge. The experiential paradigm “explores the landscape experience phenomenologically” (p. 87) in an approach which is deliberately less structured than others and where the persons are active participants (Southwell, 2004). The importance lies in the definition of the value of an environment as it is experienced by the individual, and not as it is documented by the researcher, therefore on the subjective experience (Southwell, 2004). The approach seeks to elicit the understanding of the experience as it happens, without attempting to be analytical at that stage, and avoiding preconceived notions that may distort the raw experience (Southwell, 2004). It also seeks to draw on personal reflections, shared individually or within a group, which were not possible in this case due to the nature of the participants and the ethical considerations governing the research. This is compensated for by adopting an existing accredited mapping methodology for recording the behavior of people with dementia without interfering with their activities or causing any distress. This is discussed in Tool 1.

### Tool 1—Behavior category codes tables

“*Both the according of personhood, and the failure to do so, have consequences that are empirically testable”*

Kitwood, 2019, p. 7

Tool 1, and the basis of the research methodology, is developed as a modification based on the Dementia Care Mapping 8th (DCM) framework. Dementia Care Mapping is an internationally recognized practice development intervention method that was developed over 20 years ago by the Bradford Dementia Group, specifically by Tom Kitwood, and has been reviewed and revised regularly over the years (University of Bradford, 2016). DCM is defined as an observational tool designed for use in practice assessment, quality monitoring, organization of care and staff development (University of Bradford, 2016). The Alzheimer's Society website introduces DCM as an observation method that is “a way to measure the experience of dementia” and provide “continuous quality improvement in providing person-centered care” (Alzheimer's Society, 2022, July 21).

Kitwood had developed DCM further as a mechanism for assessing the impact of the psychological and social context of people living with dementia (Brooker, 2019), by taking the standpoint of the person with dementia and using a balance of empathy and observational competence (Kitwood, 2019). Eventually DCM provided the groundwork for an observational tool custom-designed for the regulation and monitoring of health and social care environments in the UK (Brooker et al., 2007, as cited in Brooker, 2019).

Kitwood's enriched model of dementia asserts that the cumulative outcome of a person with dementia's actions, thoughts and feelings is composed of neurological impairment, health and physical wellbeing, biography, personality and social psychology (Kitwood, 1993, as cited in University of Bradford, 2016). As discussed in the review of the literature informing this study, a person is also deeply affected by her immediate environment, even more so a person experiencing cognitive decline. Therefore, it is inconceivable to assess the behavior and wellbeing of a person with dementia, without due consideration and assessment of the environment within which such actions are unfolding. This would hold also in the case of an assessment investigating exclusively a given model of care. The carers are acting within a specific context which is in/directly affecting the quality of their care provision, whether they are aware of it or not. Here it may seem trivial, albeit essential, to draw a simple analogy: a specific staff member caring for a specific person with dementia will perform differently in two different environments, where one care setting affords better quality overall in comparison to the other. We respond to our surroundings constantly, in a way we are generally unaware of and treat as automatic (Ballantyne, 2007).

For this reason, the portion of the DCM framework which is appropriately applicable to this study is that which deals with the assignment of behavior category codes to the activity of the person with dementia and the respective mood and engagement appraisal. Residents' self-assessed quality of life is strongly linked with mood, with studies suggesting that improved mood would lead to increased quality of life, even in people with severe dementia (Hoe et al., 2006, as cited in Pollock and McNair, 2012). Engagement in turn also affects mood and is driven by a broad range of environmental factors including “the physical, social, psychological, and emotional environment, as well as the experience of nature” (Bossen, 2010, p. 19). The value of positive engagement and activity elicited by the breadth of environmental factors available to the person with dementia is also broadly accepted (Perin, 1970; Kaplan and Kaplan, 1989; Passini, 1992; Cohen-Mansfield and Werner, 1995; Tyson, 2002; Rappe and Linden, 2004; Chalfont, 2005; Marshall, 2006; Zumthor, 2006; Chalfont, 2008; Topo and Kotilainen, 2009; Gehl, 2011; Brawley, 2012; Morgan-Brown and Chard, 2014; Cohen-Mansfield et al., 2015; Kitwood, 2019; Bowes and Dawson, 2019).

Therefore, DCM was used for this tool to assess the wellbeing of residents by noting their mood and engagement as well as behavior for each time segment. The maps for each individual, for each time period, were also used in the generation of group maps summarizing the behavior of the whole group. These group maps follow the DCM Excel formulae to denote the following:

- The general wellbeing of the group in terms of mood and engagement values, across the DCM range −5 to +5, against percentage of time.
- The group behavior profile, denoting the behavior categories engaged in, against respective percentage of time, for the mapping period.
- The individual wellbeing score, which gives an overview of the average wellbeing recorded for each participant, allowing for a comparative overview of the participants for the mapping period.

The study adopted the standard DCM eight tables used for coding the behavior observed, and for assigning the appropriate mood and engagement value to each action.

**Figure 4:**
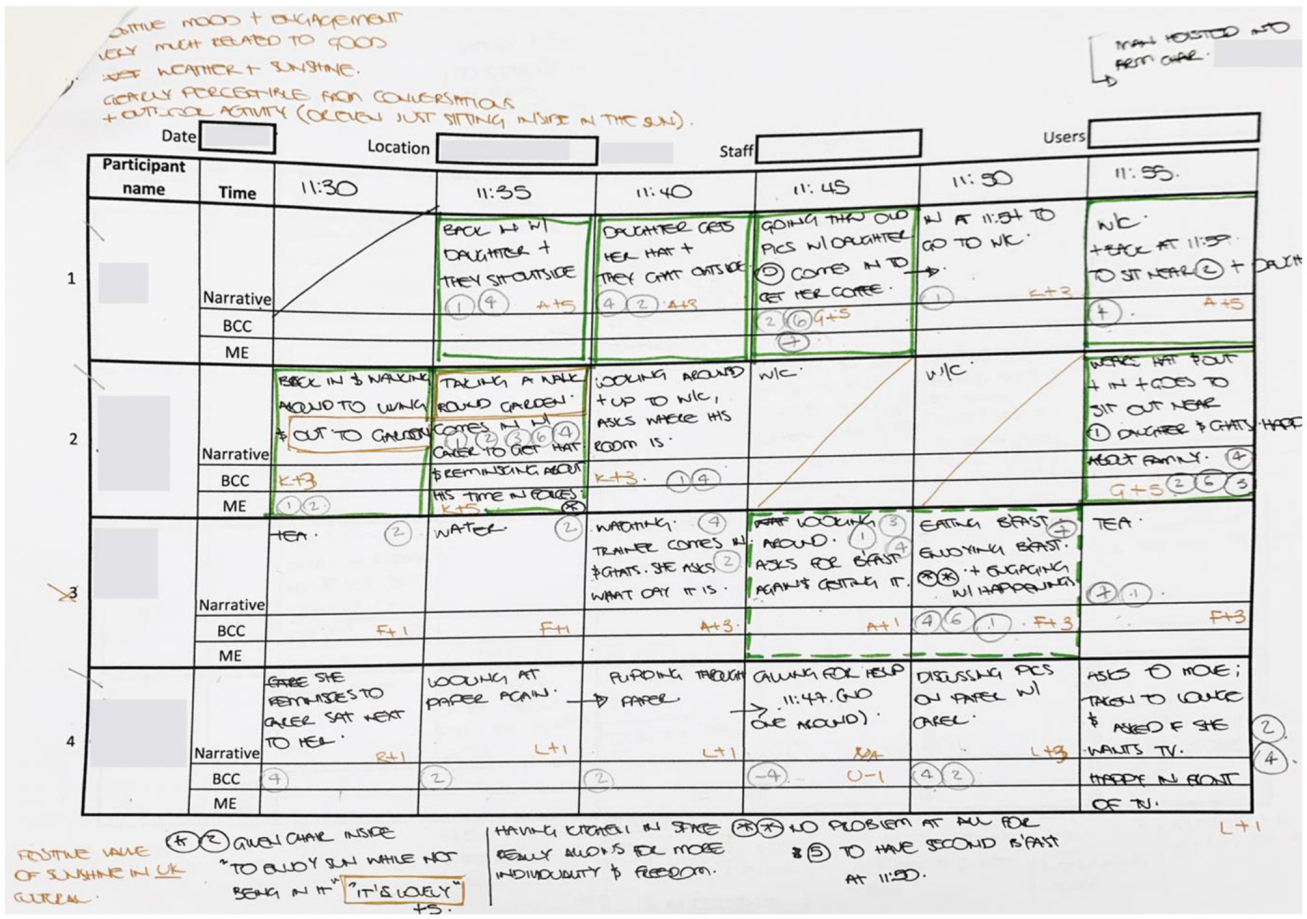
Sample hand-written mapping sheet. Source: Author.

**Figure 5:**
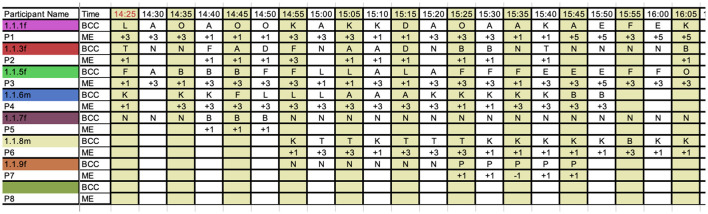
Sample DCM data sheet. Source: Author using standard DCM data sheets.

Handwritten field notes were also used to supplement the standard DCM data. The general field notes included the weather and temperature on the day as well as any general conditions worth noting, such as background music playing, an organized activity, cultural or religious event, etc., which was likely to influence the behavior of the residents. The more specific field notes included the individual identifier for each resident, a short description of their activity, descriptions of the immediate environment and possible inter-relations between persons in the space, be they residents, carers or family members. The field notes were written in short during the actual observation, then completed where necessary thereafter, Figure 4 shows a typical set of notes taken during an observation period, including superimposed notes taken after the session. This includes overlapping data from Tool 1 and Tool 2, for each time segment, that was then extracted for the respective tools. The notes for Tool 1 were coded following the DCM system. All codes were digitally tabulated using Microsoft Excel, as per sample in Figure 5. Each letter code in Figure 5 represents a pre-defined behavior as per standard DCM assessment guidelines.

### Tool 2—Response charts

“*The goal of life quality treatment is not only to maintain positive mood, but also to maintain emotional stability around social norms—recognising and dealing with other people—and personal norms—maintaining a sense of self.”*

Zeisel and Raia, 2000, p. 5

This tool follows on from the field notes written for tool 1. The active observation process sought to capture a more extensive data set that considered engagement with the site-specific factors governing each care environment following a more holistic ethnographic approach. The characteristics of ethnographic work are consistent with those of the constructivist (qualitative) strategy. These include “a holistic exploration of a setting, context-rich detail, reliance on unstructured data, a focus on a smaller number of cases, and data analysis that emphasizes the interpretation of ‘the meanings and functions of human action”' (Groat and Wang, 2013). Indeed, “place” becomes part of the process when quality time is invested in examining the site to understand how and why different spaces are used (Chalfont, 2008).

Therefore, the richness of events occurring during the observation period in the pilot study led to the design of a further tool against which to code, assess and display them. The most common themes occurring during the pilot study were extracted and the first version of the tool was developed. Following the first data collection set, the tool was reviewed and tweaked in the way different behaviors were assessed, then re-assessed again upon completion of the first Fieldwork site.

**Figure 6:**
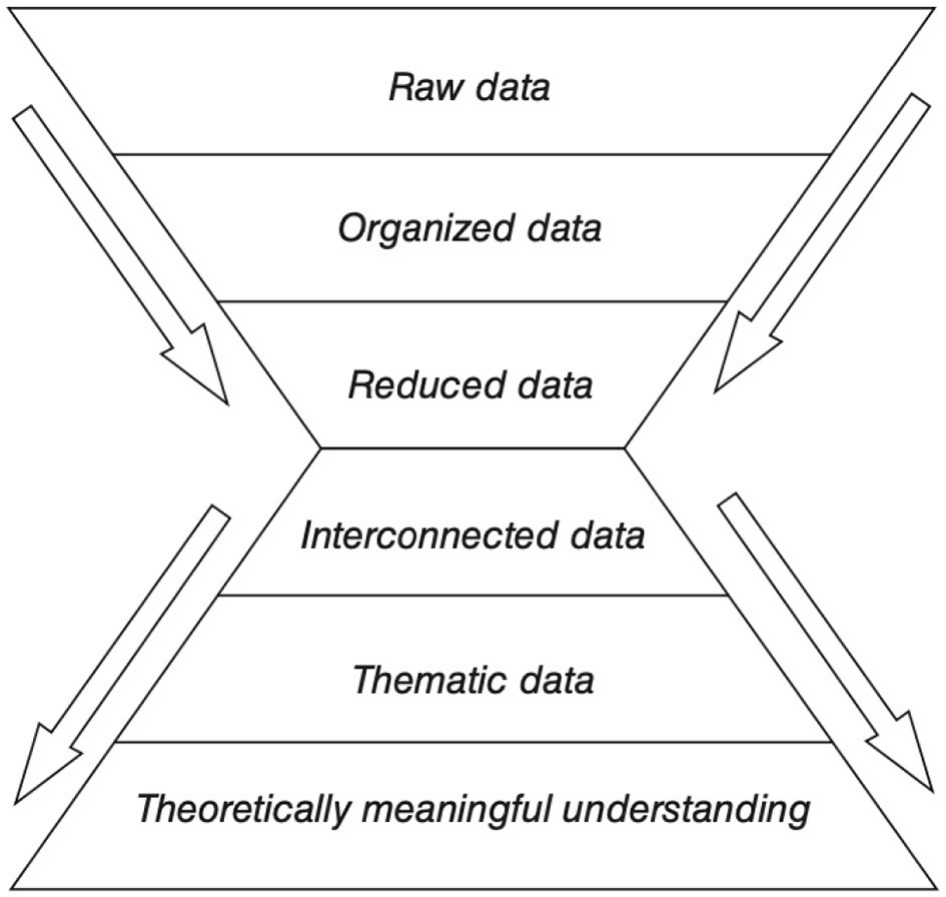
Drilling and abstracting out qualitative data. Source: O'Leary (2010), cited in Groat and Wang (2013, p. 246).

The coding process was reviewed at least three times over for each observation period to ensure that the system applied was as consistent as possible within the parameters of the interpretative phenomenological approach followed, particularly in the ways similar behaviors were analyzed and coded. O'Leary's (2010, as cited in Groat and Wang, 2013) system of drilling in and abstracting out was applied in the analysis of the data collected, where the system of six steps proposes an iterative management of data while maintaining the multifaceted qualities of the phenomenon under investigation (Figure 6).

This tool is developed by overlaying three environment-driven layers onto the standard DCM Mood and Engagement graph, resulting in a total of four data layers superimposed on each chart. The chart format proposed was also developed through an iterative process that interpreted the data against a set of defined parameters for each cycle.

**Figure 7:**
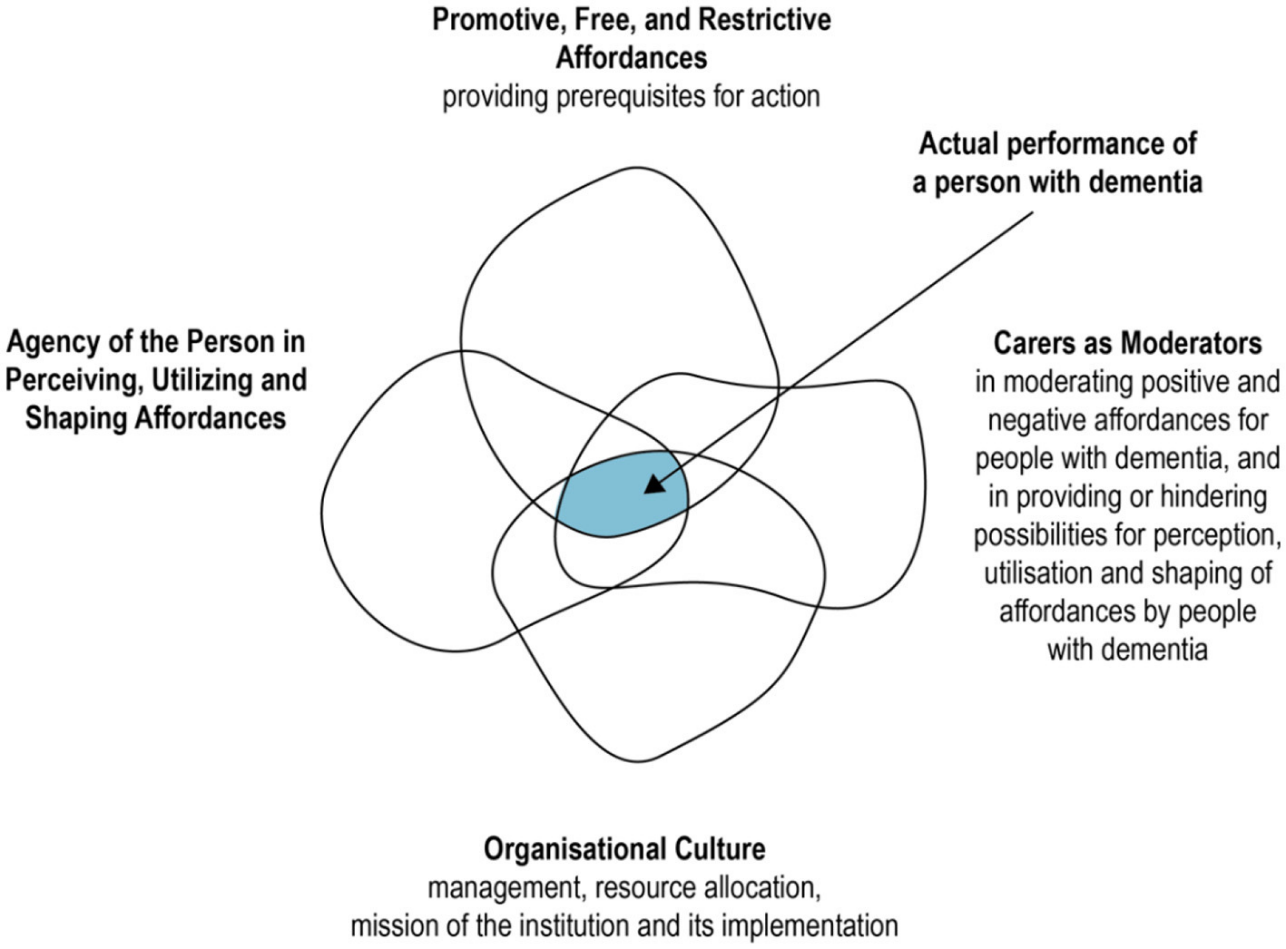
Framework depicting the performance of a person with dementia in residential care. Source: Author, adapted from Topo et al. (2012).

These charts, referred to as *response charts* are created for each user in each location and for each observation period, derived from the data maps created during the respective fieldwork. The following are the layers represented in the response charts:

Layer 1: **Mood and engagement**. Base layer of DCM Mood and Engagement graph extracted for each user, showing positive and negative mood and engagement across a scale of −5 to +5.
*Red continuous line on chart—right axis. See* *Figure 5*.
Layer 2: **Connection to outdoors**. Identifying variation between positive and negative mood and engagement which happens indoors and outdoors. Here ‘connection to outdoors' is deemed to exist not only when the person is physically outdoors, but also when the person is sitting by a window, looking out toward the outdoors or references the outdoors or other places outside the care environment, in conversation with other users and/or staff. Therefore, behavior occurring along the indoor/outdoor threshold of the building is also signified by this layer.
*Green markers on chart—right axis. See* ***Figure 13***.
Layer 3: **Noting exit-attempts**. This involves adding more value to the act of walking and distinguishing ‘pacing' from a leisurely stroll, while attempting to define environmental stressors and enhancers that may be prompting such behavior. Documentation of exit-seeking behavior (Martino-Saltzman et al., 1991; Lai and Arthur, 2003; Killick, 2006; Marshall, 2006; Stokes, 2006) proved useful in accomplishing a more comprehensive image of the experience of the care environment, particularly in identifying the different cues that may have prompted such behavior, signifying that it is not exclusively induced by the dementia.
*Orange markers on chart—right axis. See* ***Figure 13***.
Layer 4: **Documenting environmental elements affording behavior**. The concept of affordances as defined by Gibson (1979/2015) and the human perception of such affordances in the daily experience of a person with dementia is central to this methodology. This layer of the response charts attempts to capture this negotiation by recording the behavior of the individual in this context during the same naturalistic observations that are informing the other layers of the charts. The focus of this layer is on the potential and actual performance of the person with dementia, as informed by the context of the dementia care environment. The care environment referred to here encompasses the potential affordances available to the person, the carers, the organizational culture and the agency of the person, brought together in a relationship defined in Figure 7 as adapted from Topo et al.'s (2012) study. It is important to note that the set of affordances are not fixed and objective, but in relative flux, conditioned by the perception of the individual and their availability to other participants within the shared context; “When the environment changes as a result of the shaping of the affordances ... the set of potential affordances of the environment expands” (Kytta, 2004, p. 181).

This layer also accounts for physical traces of behavior that exist in the physical environment and have the potential of shedding light on the usage of the space. Zeisel (2006) argues that “traces clarify their context and are clarified by them” (p. 179), therefore the set of affordances offers the potential to be managed, affected and altered by the participants.

The spectrum of environmental elements that may be affording respective behaviors in the residents with dementia have emerged iteratively through the pilot study and first phases of the fieldwork observations and have been organized thematically as follows:

1. *Physical—Immovable:* general physical environment concerned with the fixed elements including spatial layouts, materiality, garden elements, fixed seating, fences, exit points, physical accessibility, paths, signage, etc.
2. *Physical—Movable:* objects and items within the physical environment that may be moved, including hat/coat stand, cutlery and crockery, newspapers, frames, TV, handbags, books, chairs, soft furnishings, etc.
3. ‘*Physical'—Intangible:* affordances affecting the senses, including the smell of food or plants, the sound of music, a doorbell or phone, the sense of cold or warmth etc.
4. *Social:* interaction with care staff, family members, guests/visitors and other residents in the care environment, etc.
5. *Cultural:* religious artifacts and rituals, national holidays/feasts and rituals, cultural habits and rituals, rituals in food preparation, etc.
6. *Food:* food is recorded as a separate entry as it is generally a purveyor of positive mood and engagement and is a unique affordance in the type of behavior it affords. In fact, at times food has also been noted to be used by caring staff to shift specific unfavorable behavior.

By including the affordances on the same chart as the mood and engagement for the individual, the extent of positive or negative effect of the affordance on the individual is also immediately noted.

This data is presented in a stacked area chart in shades of blue. The darkest shade represents the more tangible affordances and gradually fades to a light shade of blue representing the less tangible ones. Food is marked in yellow as a unique affordance. Left axis.

The response charts were developed digitally using Excel and Adobe Illustrator, an example of which is shown in the sample outcome.

### Tool 3—Travel route maps

This tool proposes the creation of a map of the walking route per person per observation period, in order to identify which spaces afford more use and meaningful engagement. Apart from considering the architectural elements of the environment, following the theorem of nodes, paths, landmarks, edges and districts (Lynch, 1960), consideration is given to the environmental meaning these may hold, whether functional or sensorial (Passini, 1992). For people with dementia the imageability or legibility of the environment, as central to a person's adequate functioning, is more likely to be process-oriented and sensory-driven (as per Gibson's (1979/2015) definition of environmental legibility), thereby making the physical environmental features even more relevant for this user group.

The maps allow for the identification of the following parameters related to the movement across the care environment:

- Most used paths and places.
- Movement per time of day.
- Classification of walking and pacing.
- Average distance walked per person, with the possibility of analyzing this also in terms of time spent walking.

**Figure 8:**
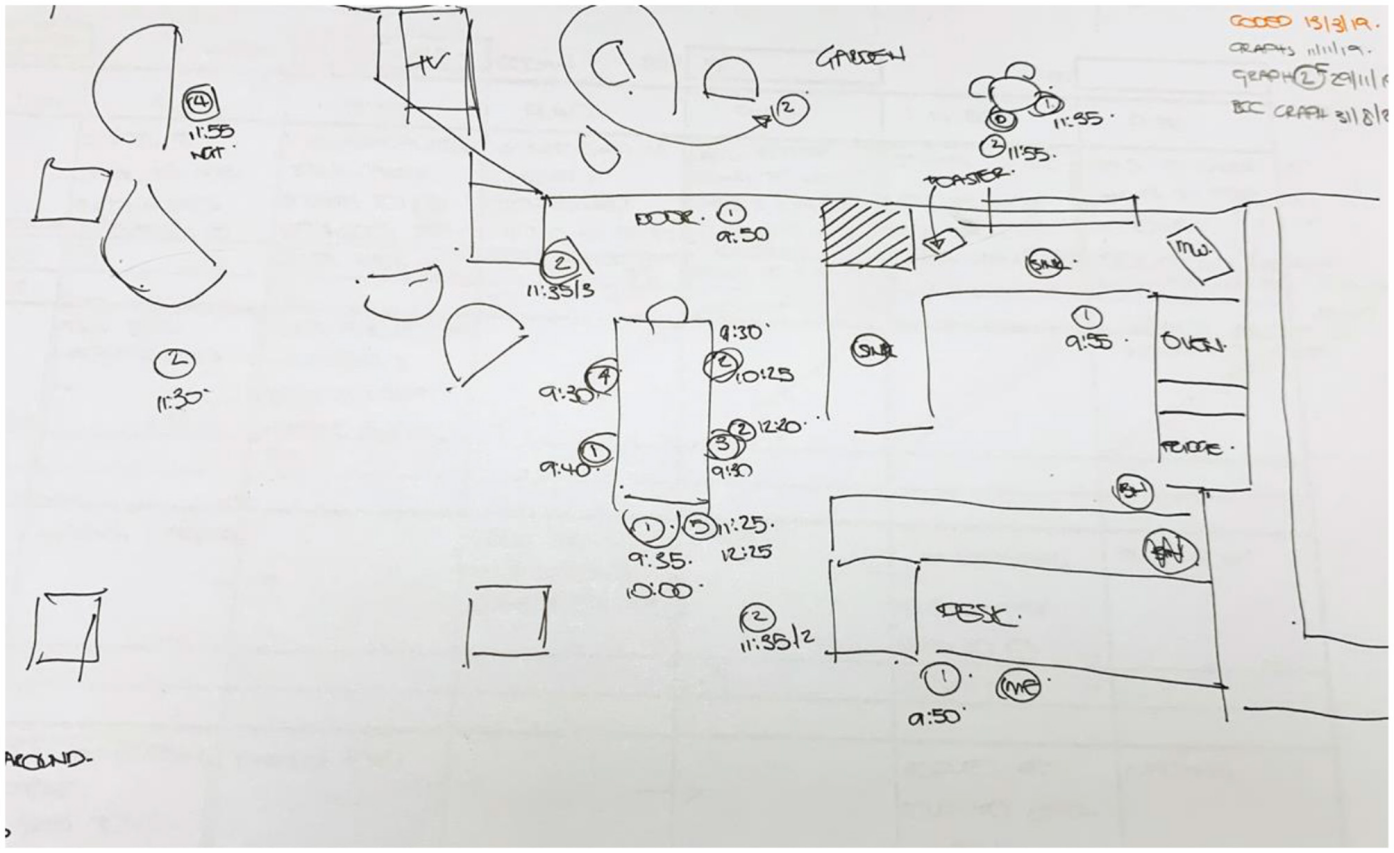
Sample route working diagram. Source: Author.

Through the parallels between this tool and the previous tools, it is also possible to correlate the mood and engagement of the person while walking, movement patterns in relation to weather (particularly relevant to outdoor walking), movement patterns in relation household activities, affordances experienced throughout the movement, etc. A typical fieldwork sheet showing the mapping for the travel routes is shown in Figure 8 (this figure is part of a number of working sketches that are used to locate the participants at different instances, an example of the final map is shown in the sample outcome). The fieldwork sheets were transposed digitally into travel route maps using AutoCad and Adobe Illustrator.

### Tool 4—Cultural setting diagrams

“*Architecture involves this cultural aspect of buildings, which can range from something very personal and idiosyncratic to something that everyone seems to agree upon. We are shaped by the culture that we grow up in, and by the culture in which we participate, whether we think about it or not, and most of the time we don't think about it at all.”*

Ballantyne, 2007, p. 19

The cultural setting diagrams were designed to capture a particular moment which signifies an individual or group response to a given affordance or set of affordances, a peak or trough in the charts of the previous tools, a particular reaction that draws further attention. The sketch is used as a tool that condenses a large amount of information while drawing in the reader to the real-life experience of the dementia care setting under investigation.

The sketches were drawn freehand then scanned and enhanced using Adobe Photoshop.

### Toolkit application

The toolkit methodology described above has been applied to several international fieldwork sites.

The aim is for the toolkit to be applied in its entirety to a given site, with data drawn and analyzed separately for each site, “because a context sensitive study should not combine data from two clearly different countries” (Kytta, 2004). Each context is therefore treated as its own entity, following an effort to maintain each site detached from comparison with other sites in a common fieldwork pool.

The toolkit proposed allows for an in-depth study of the conditions of each unique site which allows for an understanding of the social and cultural, as well as the physical environment which exist together as an inseparable entity (Kytta, 2004), the adaptation to which is dependent on the person's individual characteristics (Bronfenbrenner, 1992). The toolkit opens a window onto the daily life of a person with dementia residing in a long-term care setting by providing data-rich “stills” of the experience of the person in the way she perceives her immediate physical, social and cultural environment.

## Reviewing the quality of the proposed methodological framework

The quality of the research strategy proposed has been reviewed in terms of the quality standards for naturalistic inquiry proposed by Egon Guba (1981, as cited in Groat and Wang, 2013). Groat and Wang (2013) describe the key themes of Guba's criteria “for assessing trustworthiness... [as] the recognition of multiple realities, as opposed to a single reality; the assumption that generalizations are not necessarily possible in all instances; the understanding that a research design may emerge as the research proceeds; and the belief that the researcher and the respondent influence and are influenced by each other” (p. 84). This extract represents the essence of the proposed strategy, process followed and role of the researcher in its entirety.

The four standards of quality for qualitative research proposed by Guba are credibility, transferability, dependability and confirmability (Groat and Wang, 2013). The methodological framework proposed is therefore reviewed in the context of these four standards as follows:

**Credibility**—This denotes a more holistic approach to the problem, and the requirement “to establish truth value by taking into account the natural complexities inherent in the situation... being studied” (Groat and Wang, 2013, p. 84). Groat and Wang (2013) conclude that truth is demonstrated by triangulation and member checks. For this research, triangulation is achieved by the combination of data collection strategies. Member checks, for checking data checks and interpretations (Groat and Wang, 2013), are fulfilled through the conversations with people with dementia and their family carers, as well as the introductory interviews with the care home manager at the start of each observation period.
**Transferability**—This is equivalent to ‘generalisability' in the postpositivist paradigm and refers to the extent of applicability of conclusions to different settings (Groat and Wang, 2013). Here Guba (1981, as cited in Groat and Wang, 2013) argues that “a sufficiently ‘thick' description” (p. 85) would allow for the emergence of similarities for assessment. For this study, the volume of data collected in each of the care settings is sufficiently extensive to permit extraction of tangible themes without the risk of generalizations that may jeopardize the qualitative nature of the observations. The fact that in-depth analysis is carried out for each site, with the emergent themes being correlated across five sites, would be sufficient to assume that the common emergent themes are not unique to the specific care environments under investigation.
**Dependability**—This refers to a fundamental consistency in the data and the method of analysis, that includes any shifts or developments arising from researcher insights throughout the research process (Groat and Wang, 2013). An audit trail, as the main device for ensuring such reliability, requires thorough documentation of the way the data was collected, analyzed and interpreted (Guba, 1981, as cited in Groat and Wang, 2013). This study presented an immersive strategy, as described above, detailing the way the data was collected, the process and analysis specific to each site would then follow together with the interpretation of the data. Notes, diagrams, and journal entries are included to support the themes in the analysis and discussion, in a bid to maximize the dependability of the study.
**Confirmability**—Opposed to the researcher's objectivity, confirmability of data and its interpretations can be achieved through triangulation and the researcher's reflexivity (Guba, 1981, as cited in Groat and Wang, 2013). While triangulation involves the use of different methods and sources, as established previously, reflexivity requires the researcher to “reveal her epistemological assumptions, their influence on the framing of the research question, and any changes in perspective that might emerge during the course of the study” (Groat and Wang, 2013, p. 86). While this type of study seeks to bring to the fore the perspective of people with dementia from their point of view, such perspectives also have to be balanced with the researcher's/researchers' own, grounded in their own experiences that are activated in the interpretation of the behavior that is observed and analyzed. It is ideal to clarify the researcher's/researchers' position at the onset, as well as the developmental shifts experienced as part of the process.

## Sample outcome

The following is a sample outcome set showing the behavior, mood and engagement of a person with dementia resident in a dementia care unit in Malta. The introduction would include a general description of the unit, the context, cultural setting and physical parameters pertinent to the space. Each resident would be described in the context of the environment as well as behavior of the other residents during the observation period, therefore resulting in a thicker description of the cultural setting, and resident interactions, which is not part of the scope of this paper.

In a typical results presentation and discussion, the following sequence of data would be presented, starting with the context of the person in terms of the general behavior observed during the specific time period. The first chart is a Group WIB (well and ill-being) Profile which shows the percentage amount of time devoted to the different mood and engagement values (ME Values) for this time period. This chart is extracted directly from the DCM values and shown in **Figure 10**.

**Figure 9:**
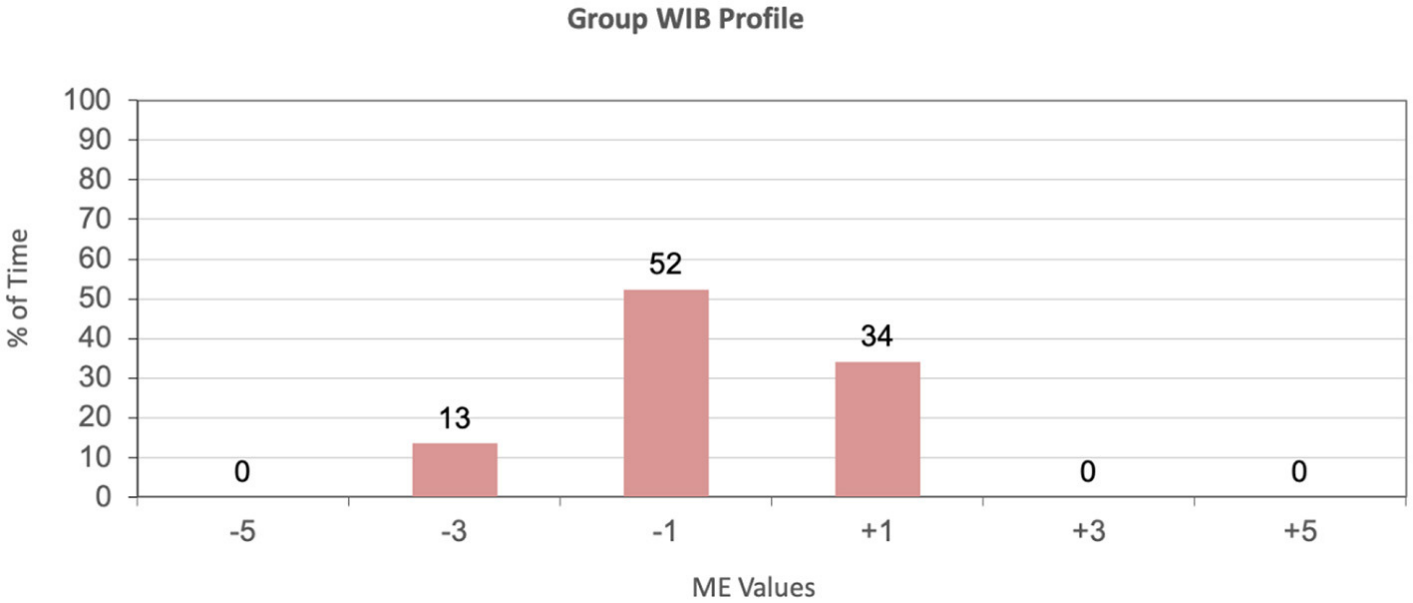
Location X_Day X_Group well- and ill-being profile.

Figure 9 shows that the residents expressed ill-being for most of the time (65%), with the majority of the residents' behavior denoting possible withdrawal and smaller signs of negative mood and engagement (−1), and a few episodes of considerable negative mood or displeasure (−3). The highest level of mood and engagement is only at the neutral level (+1), therefore no visible signs of positive mood and engagement were recorded for any of the residents during this observation period. This implies that the mean mood and engagement value for this time period is also negative, at −0.6.

**Figure 10:**
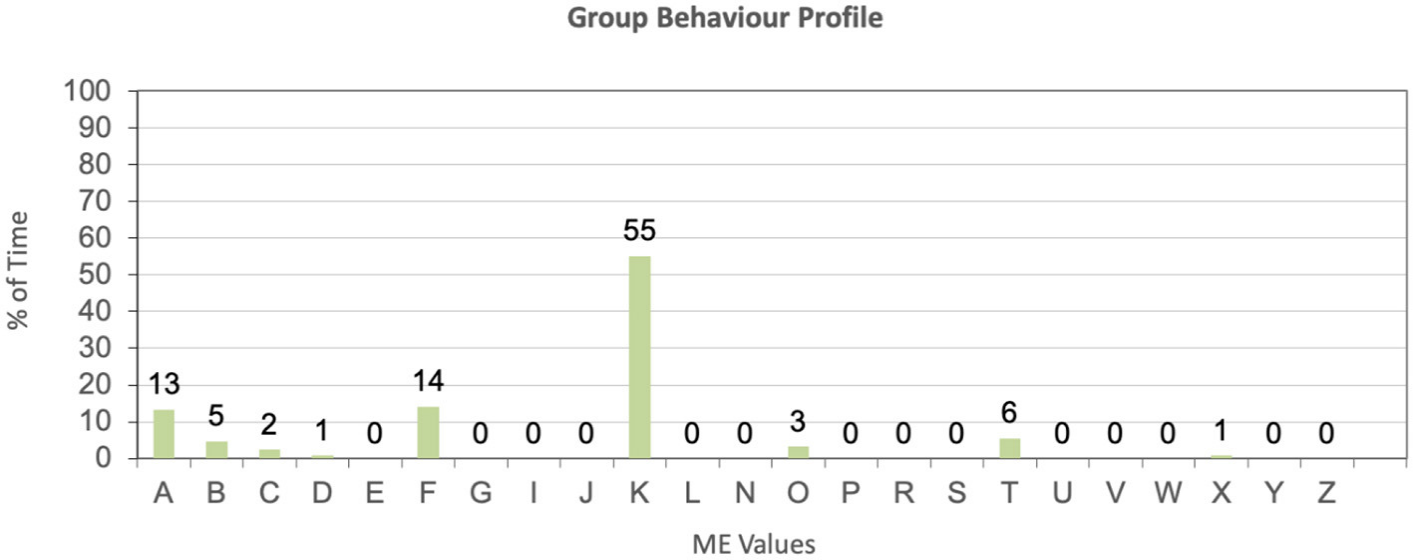
Location X_Day X_Group behavior profile.

The next chart is a Group Behavior Profile, which is also extracted directly from the DCM values. This observation period is characterized by considerable amounts of walking, as the behavior that covered over half of the cumulative time for this observation period. Figure 10 shows the time spent on walking (K) in the context of the other behaviors observed.

Other common behaviors are food (F), related to the residents enjoying their lunch, as well as articulation and communication (A), which is a relatively low value at 13%. Here it must be noted that further communication would have taken place during the observation period, but where this is not the predominant activity, it would not be noted as such, as per DCM 8 guidelines.

**Figure 11:**
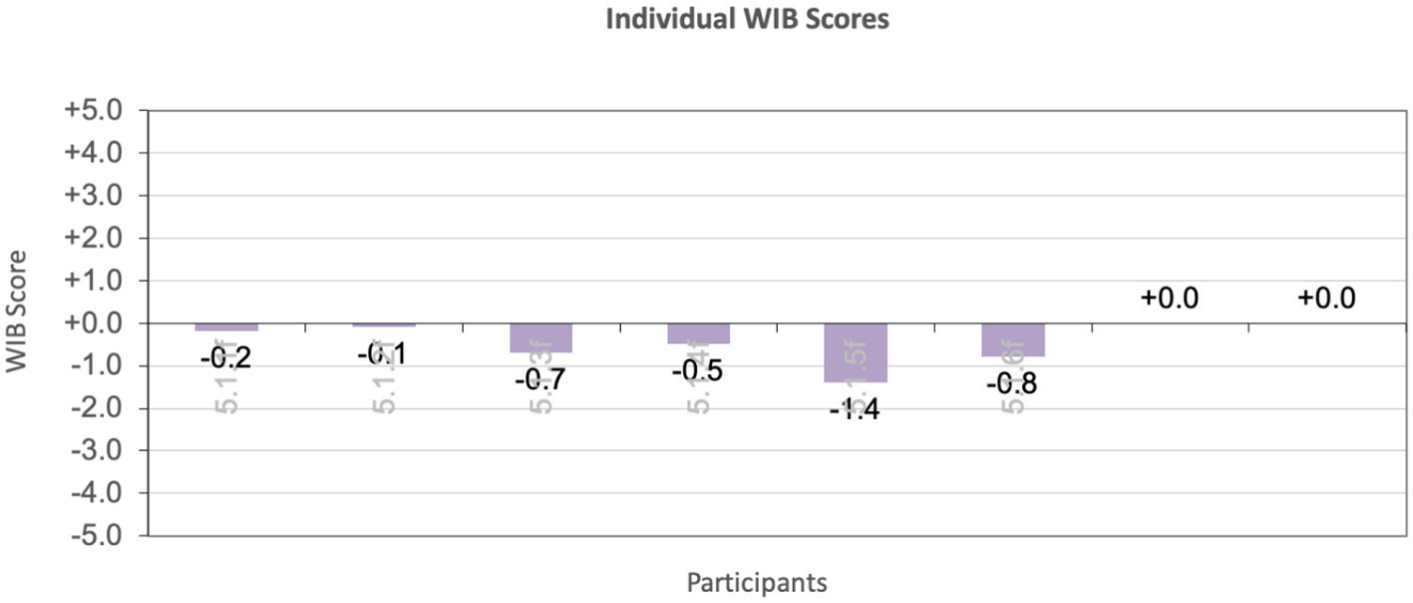
Location X_Day X_Individual well- and ill-being scores.

In fact, an amount of conversation took place simultaneously with the walking, where the women were observed walking and discussing places they had to get to, things they had to do and catch up with. Despite walking being generally regarded as a positive, healthy activity, it was associated with negative behavior in this case due to the typology and style of the walking, as well as the comments of some of the women walking. In terms of the individual wellbeing of the residents, Figure 11 shows that all the residents scored negative well- and ill-being scores during this observation period, with resident 5 scoring the worst level of ill-being at −1.4, and resident 3 and resident 6 following at −0.7 and −0.8 respectively. Resident 5 was continually asking to go out and go home. She was observed attempting to exit the ward, then asking one of the visiting doctors how he managed to get in, and trying to follow another member of staff out. She was particularly vociferous about her wish to go home, expressing clear anger and frustration when she attempted to leave or noticed a staff member arriving or leaving, and did not make it to the door in time.

It was noted that several women walked, more commonly in a group led by resident 1, who at different instances would draw in different women and encourage them to join her, while inciting them to walk faster as they were late, either to purchase the bread, other food items, or because someone was waiting for them.

**Figure 12:**
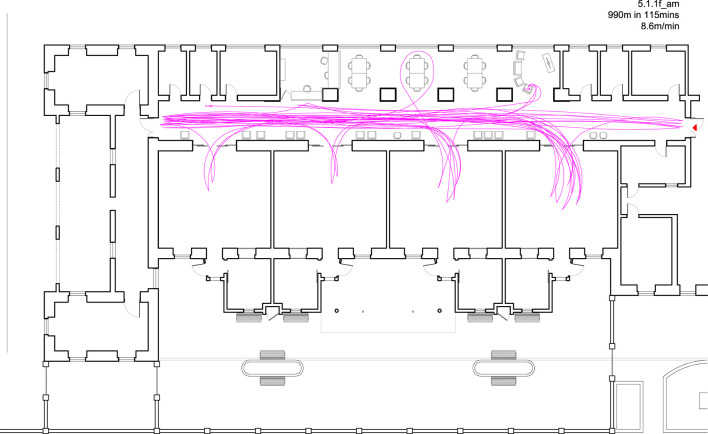
Location X_Day X_Travel route for resident 1.

Traditionally in Malta, bread would be delivered through the streets by cart, then by van, at a specific time daily, in some locations twice daily, providing fresh bread to go with each meal. The behavior of resident 1 is therefore conceivable as highly meaningful to her in the context of her previous lifestyle, where as a homemaker, ensuring her kitchen was always well-stocked, and maintaining a good relationship with her vendors was central. Resident 1 was observed to be relatively fit and managed her walking comfortably, covering a considerable distance of 990 m in 115 min. Figure 12 shows her travel route for this observation period, where she walked at an approximate speed of 8.6 m/min. She was observed walking from one exit point to another in an attempt to get out and get to her shopping on time, also going to the other communal bedrooms regularly as she tried to make her way out. The fact she went to all the communal bedrooms, and not just her own, may suggest that she was either looking for an exit or she was confused by the physical similarity in all of the rooms. While walking, she continually repeated her need to arrive on time, at times looking stressed as she recruited other residents along the way, to help her. While many of the other residents obliged and joined in to walk with her, they didn't all enjoy the same agility, clearly struggling to keep up with her at times. Resident 1 would then leave that particular resident and move on to another and continue walking with her.

**Figure 13:**
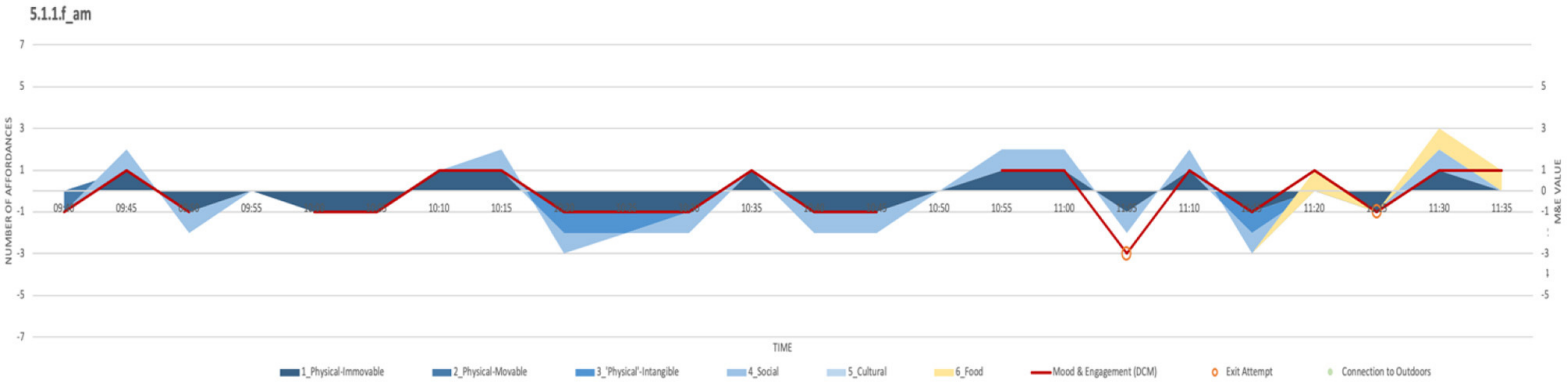
Location X_Day X_Response chart for resident 1.

The response chart sheds light on the environmental elements that may have triggered the resident's behavior throughout this observation period. The physical environment features predominantly in the chart of resident 1 as one of the women who spent most of this observation period walking and was clearly strongly informed by the environment in doing so (such conclusion may be reached when comparing the behavior of resident 1 to that of the broader group of residents during the observation period, with particular focus on the walking styles and behavior of those residents that spent the whole period walking).

Resident 1 displays a more undulating mood and wellbeing outline, governed by shifts between physical environment triggers and social ones, derived mainly from conversations with another resident who was walking with her or directed toward the other women within the space. During many of the occasions when the women reached the more commonly used of the two exits, exit attempts occurred in an almost systematic manner.

In their group and individual repetitive walking style, the women during this observation period displayed a social phenomenon of mirrored behavior (Barker, 1968) common also amongst people with dementia. Moreover, the layout of the unit meant that the residents who were not partaking in the walking, could not avoid it in any way, at times joining in if they were physically able to do so, and proceeding to follow the behavior in attempting to open the doors at the exits. This in turn resulted in frustration and distress as they followed in a cycle of attempts at all the doors in the unit. The superimposition of the walking pattern.

**Figure 14:**
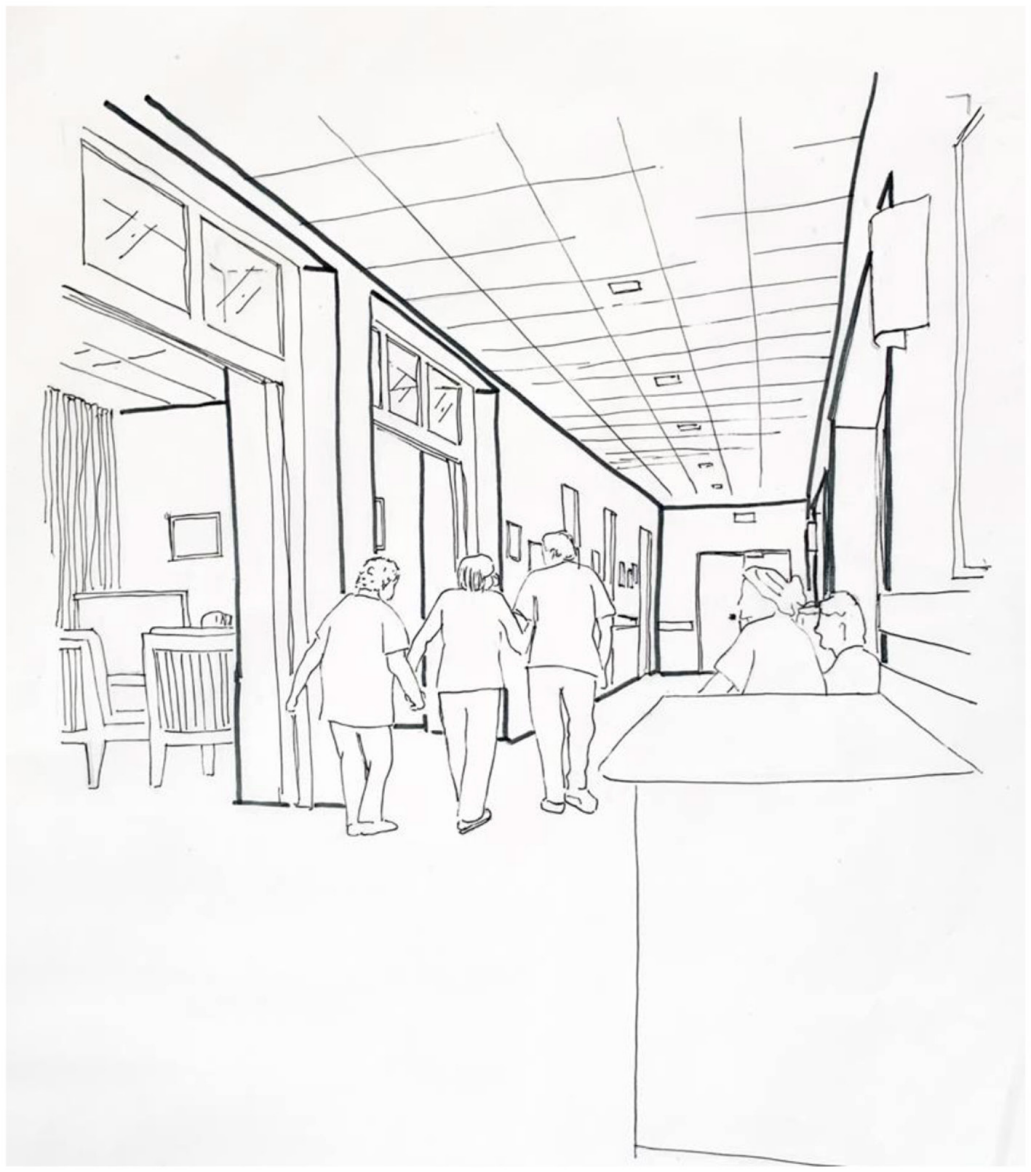
Location X_Day X_Cultural setting diagram_Residents 1, 3, 5.

Figure 14 shows a cultural setting diagram for this period, depicting three women walking hand-in-hand toward the exit. This is a snapshot of the residents that would generally be taken to emphasize the most particular or noteworthy type of behavior observed during a given observation period.

In this example, the ward was very much devoid of movable elements, except for very basic furniture and medical equipment, causing deficiencies in the potential elements available for engagement. The few instances when two residents were noted attempting to engage with a movable element, a diaper and a medical trolley respectively, the residents immediately had the items taken away from them by the care staff. The limited availability of physical movable items is also shown in the response chart summary in Figure 14.

**Figure 15:**
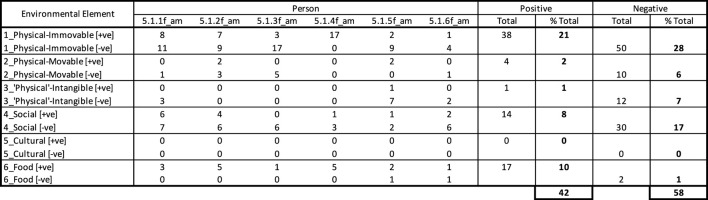
Location X_Day X_Response charts summary.

The response charts summary (Figure 15) also shows that the majority of environmental elements manifested in negative behavior, with the majority exacerbated by the physical environment, particularly the immovable elements. This refers to the physical layout, flooring typology and lack of connection to the outdoors which the residents clearly sought and requested regularly. The negative social elements in fact refer to such requests, and the extent to which they were unmet by anyone on the ward. This appears to have been difficult for the care staff, who were also subjected to performing their duties (which may have excluded allowing residents access outdoors) to the best of their skill, within a relatively challenging physical environment.

This sample depicts the behavior of only one resident in the context of her care environment for a specific time period. For each time period the method affords the collection and tabulation of data from up to 9–10 residents, providing a highly nuanced image of the perceived daily life experience of the residents in a given care environment, individually and as a group.

## Conclusion

The methodology proposed takes existing dementia mapping methods as a basis and advances them in an iterative manner as part of a broader research process and phenomenological analysis. It was developed following a thorough review of existing mapping methods and tools, where two main gaps were identified: (i) In the way such data related to the environment as experienced by the person with dementia; (ii) In the accessibility and usefulness of such data to architects and designers who are seeking guidance on how to improve the design of future care environments for people with dementia. In a highly qualitative manner, the method draws on contemporary circumstances that are both social and cultural, with an emphasis on the subjectivity of the experience of the care environment, based on the individual's perception of the affordances available to her.

The phenomenological methodology proposed exists at the threshold of the intersubjective and constructivist paradigms, eliciting deep insights and interpretations of the care environment from the point of view of the individual experience of the person with dementia and her observer relation to her immediate surroundings. This is driven by an emphasis on the natural setting, a focus on interpretation and meaning (by the observer) and a focus on how people with dementia make sense of their immediate environment.

The theory of affordances is applied in realizing the perception of the participants as they navigate the indoor and outdoor spaces of their care environment, in the analysis of those elements that effect the mood and engagement of the residents with dementia. This theory is incorporated into a comprehensive toolkit composed of four tools that are designed for a sequential application toward a holistic image of a person's experience in a specific place and time. These naturalistic observations may be further augmented with conversations with people with dementia and interviews with family and professional carers to further validate the reality presented.

## Data Availability

The raw data supporting the conclusions of this article will be made available by the authors, without undue reservation.

## Author's note

Dementia has a disproportionate impact on women, with the disease being far more common among women than men (Alzheimer's Society, 2022, November 20), and women also providing the majority of the care hours, accounting for 70% of the total care hours (World Health Organization, 2022, November 20). In acknowledging the considerable effect that dementia has on women, and therefore the larger number of women residing in care environments, the pronoun she is used throughout this paper.

## References

[B1] Alexander C. (1977). A Pattern Language. Oxford: Oxford University Press.

[B2] Alzheimer's Society (2022). What is Dementia? Symptoms, Causes and Treatment. Available online at: https://www.alzheimers.org.uk/about-dementia/types-dementia/what-is-dementia (accessed September 10, 2022).

[B3] Ballantyne A. (2007). Deleuze and Guattari for Architects. London: Routledge. 10.4324/9780203934203

[B4] Ballantyne A. (2007). Deleuze & *Guattari for Architects*. London: Routledge.

[B5] Barker R. (1968). Ecological Psychology*: Concepts and Methods for Studying the Environment of Human Behaviour*. Stanford, CA: Stanford University Press.

[B6] Bennett K. (2006). “Designing for walking: creating rich environments,” in Dementia: Walking Not Wandering, eds. M. Marshall, and K. Allan (London: Hawker Publications), 88–94.

[B7] Bossen A. (2010). The importance of getting back to nature for people with dementia. J. Gerontol. Nurs. 36, 17–22. 10.3928/00989134-20100111-01 20128524

[B8] Bowes A. Dawson A. (2019). Designing Environments for People with Dementia. Leeds: Emerald Publishing. 10.1108/9781787699717

[B9] Brawley E. C. (2012). “Designing successful gardens and outdoor spaces for individuals with Alzheimer's disease,” in Outdoor Environments for People with Dementia, eds. S. Rodiek, and B. Schwarz (Binghamton, NY: The Haworth Press), 265–284.

[B10] Bronfenbrenner U. (1992). “Ecological systems theory,” in Six *Theories of Child Development: Revised Formulations and Current Issues*, ed. R. Vasta (London: Jessia Kingsley Publishers), 187–249.

[B11] Brooker D. (2019). “On being a person; commentary by Dawn Brooker,” in Dementia Reconsidered, Revisited: The Person Still Comes First, ed. D. Brooker (London: Open University Press), 78–82.

[B12] Cerejeira J. Lagarto L. Mukaetova-Ladinska E. B. (2012). Behavioral and psychological symptoms of dementia. Front. Neurol. 3:73. 10.3389/fneur.2012.00073 22586419 PMC3345875

[B13] Chalfont G. (2005). Building edge: an ecological approach to research and design of environments for people with dementia. Alzheimers Care Q. 6, 341–348.

[B14] Chalfont G. (2008). Design for Nature in Dementia Care. Bradford Dementia Group Good Practice Guides. London: Jessica Kingsley Publishers.

[B15] Cohen-Mansfield J. Dakheel-Ali M. Marx M. S. Thein K. Regier N. G. (2015). Which unmet needs contribute to behavior problems in persons with advanced dementia? Psychiatry Res. 228, 59–64. 10.1016/j.psychres.2015.03.043 25933478 PMC4451402

[B16] Cohen-Mansfield J. Werner P. (1995). Environmental influences on agitation: an integrative summary of an observational study. Am. J. Alzheimers Care Relat. Disord. Res 10, 32–39. 10.1177/153331759501000108

[B17] Cooper Marcus C. (2006). House as a Mirror of Self. Philadelphia, PA: Nicolas-Hays.

[B18] Creswell J. W. (2007). Qualitative Inquiry and Research Design: *Choosing Among Five Approaches*. London: Sage.

[B19] Cullen G. (1961). The Concise Townscape. New York, NY: Architectural Press.

[B20] Dementia Services Development Centre (2008). Design for People with Dementia. Audit Tool. Stirling: University of Stirling.

[B21] Easton T. Ratcliffe J. (2020). “The economics of design,” in World Alzheimer Report 2020: Design, Dignity, Dementia: Dementia-related Design and the Built Environment, eds. R. Fleming, J. Zeisel, and K. Bennett (London: Alzhiemer's Disease International), 162–171.

[B22] Gehl J. (2011). Life Between Buildings. Washington, DC: Island Press.

[B23] Gibson J. J. (1979/2015). The Ecological Approach to Visual Perception. Hove: Psychology Press (Original work published 1979).

[B24] Groat L. Wang D. (2013). Architectural Research Methods, 2nd Edn. Hoboken, NJ: John Wiley and Sons.

[B25] Hyden L.-C. (2014). Cutting Brussels sprouts: collaboration involving persons with dementia. J. Aging Stud. 29, 115–123. 10.1016/j.jaging.2014.02.004 24655679

[B26] Judd S. Marshall M. Phippen P. (1998). Design for *Dementia*. London: Journal of Dementia Care.

[B27] Kaplan R. Kaplan S. (1989). The Experience of Nature*: A Psychological Perspective*. Cambridge: Cambridge University Press.

[B28] Killick J. (2006). “More than a mile: encounters with people walking,” in Dementia: Walking not Wandering, eds. M. Marshall, and K. Allan (London: Hawker Publications), 18–20.

[B29] Kitwood T. (2019). “On being a person,” in Dementia Reconsidered, Revisited: The Person Still Comes First, ed. D. Brooker (London: Open University Press), 6–16.

[B30] Kontos P. (2004). Ethnographic reflections on selfhood, embodiment and Alzheimer's disease. Ageing Soc. 24, 829–849. 10.1017/S0144686X04002375

[B31] Kontos P. Miller K.-L. Kontos A. P. (2017). Relational citizenship: supporting embodied selfhood and relationality in dementia care. Sociol. Health Illn. 39, 182–198. 10.1111/1467-9566.12453 28177149

[B32] Kuliga S. Berwig M. Roes M. (2021). Wayfinding in People with Alzheimer's disease: perspective taking and architectural cognition – a vision paper on future dementia care research opportunities. Sustainability 13, 1–24. 10.3390/su13031084

[B33] Kytta M. (2004). The extent of children's independent mobility and the number of actualised affordances as criteria for child-friendly environments. J. Environ. Psychol. 24, 179–198. 10.1016/S0272-4944(03)00073-2

[B34] Lai C. Arthur D. (2003). “Wandering,” in Dementia Nursing: A Guide to Practice, ed. R. Hudson (Melbourne, VIC: Ausmed Publications), 70–82.

[B35] Lee S. Y. Hung L. Chaudhury H. Morelli A. (2021). Effects of physical environment on quality of life among residents with dementia in long-term care facilities in canada and sweden: a longitudinal study in a large-scale institutional setting versus a small-scale homelike setting. Archit. Res. 23, 19–28. 10.5659/AIKAR.2021.23.2.19

[B36] Lynch K. (1960). The Image of the City. Cambridge: MIT Press.

[B37] Marsden J. Meehan R. A. Calkins M. P. (2001). Therapeutic kitchens for residents with dementia. Am. J. Alzheimers Dis. Other Dement. 16, 303–311. 10.1177/153331750101600509 11603167 PMC10834010

[B38] Marshall M. (2006). “Perspectives on ‘wandering',” in Dementia: Walking not Wandering, eds. M. Marshall, and K. Allan (London: Hawker Publications), 11–14.

[B39] Marshall M. Pollock A. (2012). “Introduction,” in Designing Outdoor Spaces for People with Dementia, eds. A. Pollock, and M. Marshall (Greenwich, NSW: HammondPress and DSDC), 11–15.

[B40] Martino-Saltzman D. Blasch B. B. Morris R. D. McNeal L. W. (1991). Travel behaviors of nursing home residents perceived as wanderers and nonwanderers. Gerontologist 31, 666–672. 10.1093/geront/31.5.666 1778493

[B41] McColgan G. (2005). A place to sit; resistance strategies used to create privacy and home by people with dementia. J. Contemp. Ethnogr. 34, 410–433. 10.1177/0891241605275574

[B42] Merleau-Ponty M. (1945/2012). Phenomenology of Perception. London: Routledge (Original work published 1945). 10.4324/9780203720714

[B43] Morgan-Brown M. Chard G. (2014). Comparing communal environments using the Assessment Tool for Occupation and Social Engagement: using interactive occupation and social engagement as outcome measures. Br. J. Occup. Ther. 77, 50–58. 10.4276/030802214X1391696944699424614694

[B44] Nahemow L. Powell Lawton M. (1973). Toward an Ecological Theory of Adaptation and Aging. Jenkintown, PA: Philadelphia Geriatric Center.

[B45] Olsson A. Lampic C. Skovdahl K. Engstrom M. (2013). Persons with early-stage dementia reflect on being outdoors: a repeated interview study. Aging Ment. Health 17, 793–800. 10.1080/13607863.2013.801065 23701394

[B46] Orulv L. (2010). Placing the place, and placing oneself within it. Dementia 9, 21–44. 10.1177/1471301210364449

[B47] Ory Hernandez R. (2012). “Effects of therapeutic gardens in special care units for people with dementia: two case studies,” in Outdoor Environments for People with Dementia, eds. S. Rodiek, and B. Schwarz (Binghamton, NY: The Haworth Press), 117–152. 10.1300/J081v21n01_07

[B48] Passini R. (1992). Wayfinding in Architecture. New York, NY: Van Nostrand Reinhold.

[B49] Perin C. (1970). With Man in Mind. Cambridge, MA: MIT Press.

[B50] Pollock A. McNair D. (2012). “Going outside is essential for health and wellbeing,” in Designing Outdoor Spaces for People with Dementia, eds. A. Pollock, and M. Marshall (Greenwich, NSW: HammondPress and DSDC), 11–15.

[B51] Rappe E. Kivela S.-L. Rita H. (2006). Visiting outdoor green environments positively impacts self-rated health among older people in long-term care. Horttechnology 16, 55–59. 10.21273/HORTTECH.16.1.005535581909

[B52] Rappe E. Linden L. (2004). Plants in health care environments: experiences of the nursing personnel in homes for people with dementia. Acta Hortic. 639, 75–81. 10.17660/ActaHortic.2004.639.827409075

[B53] Rodiek S. Schwarz B. (2012). “Introduction,” in Outdoor Environments for People with Dementia, eds. S. Rodiek, and B. Schwarz (Binghamton, NY: The Haworth Press), 3–12. 10.4324/9780203726310

[B54] Southwell K. (2004). The Utility of Behavioural Science in Landscape Architecture. Edinburgh: University of Edinburgh.

[B55] Stokes G. (2006). “We walk, they wander,” in Dementia: Walking Not Wandering, eds. M. Marshall, and K. Allan (London: Hawker Publications), 28–32.

[B56] Sumathipala A. Siribaddana S. De Silva N. (2004). Qualitative research. Ceylon Med. J. 48, 136–139. 10.4038/cmj.v48i4.3332 15125407

[B57] Topo P. Kotilainen H. (2009). “Designing enabling environments for people with dementia, their family carers and formal carers,” in Dementia, Design and Technology: Time to Get Involved, eds. P. Topo, and B. Ostlund (Amsterdam: IOS Press), 45–49.

[B58] Topo P. Kotilainen H. Eloniemi-Sulkava U. (2012). Affordances of the care environment for people with dementia – an assessment study. HERD 5, 118–138. 10.1177/193758671200500410 23224811

[B59] Tyson M. (2002). Treatment gardens: naturally mapped environments and independence. Alzheimers Care Q. 3, 55–60. 10.1097/00132578-200201000-00026

[B60] University of Bradford (2016). Dementia Care Mapping: Process and Application. Bradford: School of Dementia Studies, Faculty of Health Sciences, University of Bradford.

[B61] Webb J. Williams V. Gall M. Dowling S. (2020). Misfitting the research process: shaping qualitative research “in the field” to fit people living with dementia. Int. J. Qual. Methods 19, 1–11. 10.1177/1609406919895926

[B62] Wilkinson H. (2002). “Including people with dementia in research: methods and motivations,” in The Perspectives of People with Dementia: Research Methods and Motivations, ed. H. Wilkinson (London: Jessica Kingsley Publishers), 9–24.

[B63] Wilkinson S. (2004). “Focus group research,” in Qualitative Research: Theory, Method and Practice, 2nd Edn., ed. D. Silverman (London: Sage), 177–199.

[B64] World Health Organization (2022) Dementia. Available online at: https://www.who.int/news-room/fact-sheets/detail/dementia (accessed September 10 2022).

[B65] Zeisel J. (2006). Inquiry by Design. Rev. ed. New York, NY: W.W. Norton and Company.

[B66] Zeisel J. Raia P. (2000). Non-pharmacological treatment for alzheimer's disease: a mind-brain approach. Am. J. Alzheimers Dis. Other Dement. 15, 331–340. 10.1177/153331750001500603

[B67] Zeisel J. Raia P. (2000). Non-pharmacological treatment for Alzheimer's disease: a mind-brain approach. Am. J. Alzheimers Dis. Other Demen. 15, 331–340. 10.1177/153331750001500603

[B68] Zeisel J. Silverstein N. M. Hyde J. Levkoff S. Powell Lawton M. Holmes W.. (2003). Environmental correlates to behavioral health outcomes in Alzheimer's special care units. Gerontologist 43, 697–711. 10.1093/geront/43.5.697 14570966

[B69] Zumthor P. (2006). Thinking Architecture. Basel: Birkhauser.

